# Host Factor Interaction Networks Identified by Integrative Bioinformatics Analysis Reveals Therapeutic Implications in COPD Patients With COVID-19

**DOI:** 10.3389/fphar.2021.718874

**Published:** 2021-12-23

**Authors:** Wenjiang Zheng, Ting Wang, Peng Wu, Qian Yan, Chengxin Liu, Hui Wu, Shaofeng Zhan, Xiaohong Liu, Yong Jiang, Hongfa Zhuang

**Affiliations:** ^1^ The First Clinical Medical School, Guangzhou University of Chinese Medicine, Guangzhou, China; ^2^ The First Affiliated Hospital of Chinese Medicine, Guangzhou University of Chinese Medicine, Guangzhou, China; ^3^ Shenzhen Hospital of Integrated Traditional Chinese and Western Medicine, Shenzhen, China

**Keywords:** COPD, COVID-19, comorbidity, bioinformatics analyses, host factor interaction networks

## Abstract

**Background:** The COVID-19 pandemic poses an imminent threat to humanity, especially for those who have comorbidities. Evidence of COVID-19 and COPD comorbidities is accumulating. However, data revealing the molecular mechanism of COVID-19 and COPD comorbid diseases is limited.

**Methods:** We got COVID-19/COPD -related genes from different databases by restricted screening conditions (top500), respectively, and then supplemented with COVID-19/COPD-associated genes (FDR<0.05, |LogFC|≥1) from clinical sample data sets. By taking the intersection, 42 co-morbid host factors for COVID-19 and COPD were finally obtained. On the basis of shared host factors, we conducted a series of bioinformatics analysis, including protein-protein interaction analysis, gene ontology and pathway enrichment analysis, transcription factor-gene interaction network analysis, gene-microRNA co-regulatory network analysis, tissue-specific enrichment analysis and candidate drug prediction.

**Results:** We revealed the comorbidity mechanism of COVID-19 and COPD from the perspective of host factor interaction, obtained the top ten gene and 3 modules with different biological functions. Furthermore, we have obtained the signaling pathways and concluded that dexamethasone, estradiol, progesterone, and nitric oxide shows effective interventions.

**Conclusion:** This study revealed host factor interaction networks for COVID-19 and COPD, which could confirm the potential drugs for treating the comorbidity, ultimately, enhancing the management of the respiratory disease.

## Introduction

The coronavirus disease 2019 (COVID-19) pandemic resulting from the highly contagious severe acute respiratory syndrome coronavirus 2 (SARS-CoV-2) has caused a dramatic increase in hospitalizations for pneumonia with multiple organ dysfunction. It’s reported that approximately 60–90% of hospitalized infected patients have comorbidities, most of which include hypertension, diabetes, cardiovascular disease, chronic pulmonary disease and so forth (Garg et al., 2020; Richardson et al., 2020). Given that Chronic Obstructive Pulmonary Disease (COPD) patients are prone to viral exacerbations (Bafadhel et al., 2011; George et al., 2014; Wilkinson et al., 2006) and the devastating impact the COVID-19 may have on the lungs, it is natural for them to fear in the context of the COVID-19 pandemic. The prevalence of COPD amongst hospitalized COVID-19 patients have been reported in many countries or regions, with estimates ranging from 0 to 10% in China (Guan et al., 2020a; Cai et al., 2020; Lian et al., 2020; Liu et al., 2020; Yan et al., 2020), 2.4–14% in New York City (Goyal et al., 2020; Kuno et al., 2020; Palaiodimos et al., 2020; Richardson et al., 2020), and 5.6–9.2% in Italy (Cecconi et al., 2020; Inciardi et al., 2020; Lagi et al., 2020). What’s more, several studies have found that pre‐existing COPD greatly increases the risk of severe disease and death in COVID-19 patients. A Chinese multicenter study involving 1590 COVID-19 patients showed that COPD carried an odds ratio of 2.681 (95% CI 1.424–5.048; *p* = 0.002) for ICU admission, mechanical ventilation, or death; 62.5% of severe cases had a history of COPD and 25% of those who died were COPD patients (Guan et al., 2020b). Feng et al. (2020) has also found significant differences (*p* < 0.001) between critically ill (15.7%) and moderate (2.3%) patients in the subgroup of COPD.

Currently, the interaction mechanism between COPD and COVID-19 remains unclear and there is little direct evidence about the management of COPD in people with COVID-19 (Halpin et al., 2021). It seems that the highly expressed angiotensin converting enzyme 2 (ACE2) receptors in the COPD airway, the SARS-CoV-2 receptor, were to blame, but evidence has not been shown yet to confer increased susceptibility or increased severity of disease (Leung et al., 2020; Yao et al., 2020). Moreover, COPD patients also feature endothelial cell dysfunction and increased coagulopathy, which may provide explanations for the increased risk of worse outcomes from COVID-19 (Kasahara et al., 2001; Minakata et al., 2005; Husebø et al., 2021).

Host factor networks, based on the integration of systems biology and bioinformatics, serves as a critical strategy for exploring viral diseases as well as non-viral diseases. On one hand, since viruses are obligate intracellular parasites and depend on the host to complete their life cycle, the goal of regulating virus replication can be achieved by changing the expression level of host factors closely related to virus survival. Thus, the identification of host factors involved in regulating the virus life cycle can help reveal the virus-host interaction mechanism. On the other hand, host factor networks can also further enhance our understanding of COPD, the complex and heterogeneous disease both in the clinical and biological aspects. For example, a series of studies on genome-scale identification of SARS-CoV-2 host factor networks reveals new insights into SARS-CoV-2 biology and inform ongoing drug development efforts (Daniloski et al., 2021; Hoffmann et al., 2021; Wei et al., 2021); Morrow and others (Morrow et al., 2015) used integrative genomics to identify host factors associated with specific COPD phenotypes and described a network-co-expression module that was related to the frequency of COPD exacerbations. Obeidat and others reported three co-expression modules (including interleukin 8 and 10 related pathways) associated with the severity of airflow limitation, which reveals novel gene signatures in peripheral blood for COPD patients (Obeidat et al., 2017). In short, host interaction networks allow the identification of subnetworks corresponding to the functional units of a living system, which can help us explore the pathophysiology of the disease from multiple levels, and provide insights for clarifying the virus-host immune interaction mechanism, identifying the host’s gene function, predicting underlying drugs and patient classification (Tan et al., 2007).

Therefore, we have adopted a strategy of integrative bioinformatics analysis to explore the host factor networks of COVID-19 and COPD comorbid diseases. Here, several online databases and bio-datasets were employed to identify the co-factors of COPD and COVID-19. On this basis, a series of biological information analyses were performed in an attempt to clarify the shared pathogenic molecular mechanism of comorbidities and to predict potential therapeutic drugs. Our results provide a new perspective of comorbidity interaction and identify host-derived therapeutic targets for COVID-19 and COPD.

## Materials and Methods

### Collection of COVID-19 and COPD-Related Genes

The data source for COVID-19/COPD consists of two parts, namely, databases and data sets. For COVID-19-associated genes, we referred the PubChem (https://pubchem.ncbi.nlm.nih.gov/#query=covid-19), CTD (http://ctdbase.org/), DisGeNET (https://www.disgenet.org/covid/diseases/summary/), baillielab net (https://baillielab.net/maic/covid19) and KEGG DISEASE (https://www.genome.jp/kegg/disease/) databases and data sets of (Grant et al., 2021; Blanco-Melo et al., 2020; Mahmud et al., 2021; Singh et al., 2021; Overmyer et al., 2021; Mo et al., 2021). Regarding COPD-related genes, we considered DisGeNET (https://www.disgenet.org/), CTD (http://ctdbase.org/) and GeneCards (https://www.genecards.org/) databases and data sets of (O'Beirne et al., 2020; Han et al., 2021; Morrow et al., 2017; Hu et al., 2018; Raman et al., 2009; Huang et al., 2019; Morrow et al., 2019; Shen et al., 2021). These selected data sets above represent different clinical samples of COVID-19/COPD, specifically including alveolar lavage fluid, lung tissue, airway and peripheral blood.

The top 500 genes of each database were gathered according to their ranking rules. The data sets were analyzed using GEO2R, R and limma package (Ritchie et al., 2015). Genes from data sets that meet the Benjamini–Hochberg adjusted *p*-values (False discovery rate, FDR) < 0.05 and |log2FC| ≥ 1 were selected as differentially expressed genes (DEGs). Subsequently, intersection genes of the two parts were selected as candidate targets for further analysis. The date of access to these websites was October 6, 2021.

### Analysis of TF-Gene Interactions and Gene-miRNA Coregulatory Network

The NetworkAnalyst tool (version 3.0, https://www.networkanalyst.ca/) (Zhou et al., 2019a) was used to evaluate the interaction of TF genes with common genes associated with COVID-19 and COPD comorbidities, as well as gene-miRNA interactions. The basic data of the TF-gene interaction network comes from the ENCODE ChIP-seq database (https://www.encodeproject.org/), using only peak intensity signals <500 and predicted regulatory potential score <1 (using the BETA Minus algorithm). The basic data of gene-miRNA interaction comes from miRNA-gene interaction data collected by miRTarbase comprehensively verified by experiments. Relevant results were visualized by Cytoscape (version 3.8.1, https://cytoscape.org/) (Shannon et al., 2003).

### Protein-Protein Interaction Analysis and Network Construction

Common host factors were uploaded to STRING (version 11.0, https://string-db.org/) (Szklarczyk et al., 2019) for generating PPIs network. Here, we set the minimum interaction score required by the PPI network to a medium confidence level: 0.4, and the *p*-value for PPI enrichment: 1.0e-16. The PPI results were analyzed and visualized through Cytoscape. And MCODE analysis of PPI network was subsequently performed and visualized through Metascape (https://metascape.org/) (Zhou et al., 2019b).

### Gene Ontology and Pathway Enrichment Analysis

We conducted gene ontology (GO) analysis and pathway enrichment analysis to characterize the biological mechanisms and signaling pathways of common host factor networks. GO biological processes and GO molecular functions are drawn by the WEB-based genome analysis toolkit webgestalt (Hu et al., 2021) (http://www.webgestalt.org/), and the KEGG pathway analysis results are generated by R and clusterprofiler (Yu et al., 2012) package. A cutoff of Benjamini-Hochberg adjusted *p*-values < 0.05 was adopted in this apart.

### Tissue Specific Enrichment Analysis of Top Genes

In this study, we used the multigene query function available on GTEx (Sun et al., 2021) (https://www.gtexportal.org/home/multiGeneQueryPage, accessed October 16, 2021) to perform tissue-specific enrichment analysis of 42 COVID-19 genes that overlap with COPD.

### Candidate Drugs Analysis

Overlapping genes were uploaded to ShinyGO (Ge et al., 2020) v0.741 (http://bioinformatics.sdstate.edu/go/) for further candidate drug prediction. Preset all available gene sets, *p*-value cutoff (FDR, adjusted in the hypergeometric test) < 0.05 and show the top 30 pathways. Finally, the candidate drugs from the STITCH database are screened out from the enrichment results.STITCH (Li et al., 2021) (http://stitch.embl.de/) is a powerful search tool for predicting drug-target relationships. In this analysis, we used 42 genes shared by COVID-19 and COPD to predict drug candidates for COVID-19 and COPD comorbidities.

## Results

### Identification of Common Host Factors Between COVID-19 and COPD

**FIGURE 1:**
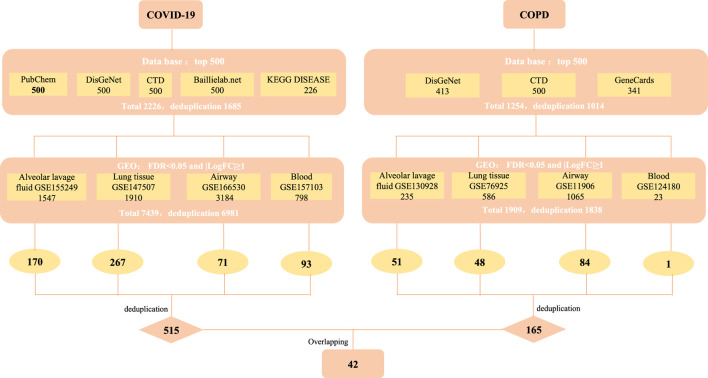
The screening process of obtaining common targets between COVID-19 and COPD. CTD: Comparative Toxicogenomics Database (http://ctdbase.org/), DisGeNET: a platform containing genes associated to human diseases (https://www.disgenet.org/), PubChem: a collection of accessible chemical information (https://pubchem.ncbi.nlm.nih.gov/), KEGG DISEASE: indicates association of genes to diseases (https://www.genome.jp/kegg/disease/), Baillielab.net:genes implicated in SARS-CoV2 infection (https://baillielab.net/), GeneCards: The Human Gene Database (https://www.genecards.org/).

We strictly screened the host factors of COVID-19. First, we searched COVID-19-related host factors from PubChem, DisGeNET, CTD, baillielab net and KEGG DISEASE, respectively. In order to improve the credibility of the data, we choose to filter the first 500 entries in each database. If it is less than 500 entries, all retrieved data will be included. Based on this, we obtained 500 host factors (after deduplication) in PubChem, CTD, DisGeNET, and baillielab net, respectively, and 226 host factors (after deduplication) in KEGG DISEASE. The host factors of the five databases are combined to a total of 1,685 after deduplication. At the same time, we also searched for COVID-19 factors in data sets that contain clinical samples. According to the screening thresholds of FDR<0.05 and |LogFC|≥1, 1,547, 1910, 3,184, and 798 differentially expressed genes (DEGs) were obtained in GSE155249, GSE147507, GSE166530 and GSE157103 after deduplication. The host factors of the four data sets are combined and deduplicated into a total of 6,981. In the end, there were 515 overlapping genes in the COVID-19 databases and data sets (Figure 1 and Table 1).

**TABLE 1:** Sources of genetic selection.

Disease	Database or GEO	Data sources	Amount of raw data	Filter condition	Amount of data after filtering and deduplication	Merge		Overlapping genes
COVID	Data base	PubChem	629	If the raw data is greater than 500, then take 500; if the raw data is less than 500, then all are included	500	1,685	515	42
		DisGeNet	1843		500			
		CTD	500		500			
		Baillielab.net	2000		500			
		KEGG DISEASE	231		226			
	GEO	Alveolar lavage fluid GSE155249	57,928	FDR <0.05 and |LogFC|≥1	1,547	6,981	—	—
		Lung tissue GSE147507	23,710		1910			
		Airway GSE166530	3,188		3,184			
		Blood GSE157103	1,054		798			
COPD	Data base	DisGeNET	448	If the raw data is greater than 500, then take 500; if the raw data is less than 500, then all are included	413	1,014	165	—
		CTD	53,814		500			
		GeneCards	341		341			
	GEO	Alveolar lavage fluid GSE130928	54,675	FDR <0.05 and |LogFC|≥1	235	1838	—	—
		Lung tissue GSE76925	32,831		586			
		Airway GSE11906	54,675		1,065			
		Blood GSE124180	31,786		23			

Similarly, to determine the host factors of COPD, we searched DisGeNET, CTD, and GeneCards to get the top 500 genes of these databases. After combining the database genes and deduplication, a total of 1014 COPD host factors were gained. In addition, we also supplemented the COPD host factors in the data sets and got 235, 586, 1,065 and 23 DEGs in GSE130928, GSE76925, GSE11906 and GSE124180 respectively. A total of 1838 host factors were obtained after merging and removing duplicates. Finally, we combined the host factors obtained by the two methods, and selected overlapping genes as the disease host factors for COPD, a total of 165. Additionally, after collecting data from the COVID-19 and COPD datasets, we sorted out the overlapping genes between different tissues, as shown in Table 2. At the same time, in order to understand more intuitively which genes are included in each database or data set, we have also traced the source distribution of 42 genes (see Supplementary Table S1).

**TABLE 2:** Overlapping genes in different tissues.

Gene source	Overlapping genes
Alveolar lavage fluid	MMP2, MMP7, RTN1, S100P, SLC22A4, RNASE6, EPS8, PRKCB, TIMP3, HS3ST1, GCLM, RASSF5, AFAP1L1, MERTK, MCOLN2, SPRY2, PLXNC1, CHST13, IFITM2, BNIP3, AOC3, CDK6, ANKRD22, SCD, SPP1, SECTM1, OSM, SPRED1, IGFBP2, GALM, GCH1, TNS1, SNCA, SLC26A11, TRERF1, SOCS3, ZC3H12C, CCL2, DFNA5, MMP12, FLT1, IFITM3, MARCKS, FAM198B, CYTL1, ADAM28, VNN1, MCOLN3, RASSF2, SLC20A1, ISG20, TRPC6, CADM1, TMEM163, SERPINE1, VCAN, SLC39A8, RASAL2, HS3ST2, CD84, SH3RF1, LINC01010, MLLT11, CYBRD1, GATM, FAM101B, AKT3, CYP1B1, XYLT1, ACKR3
Lung tissue	NOL8, TLR1, SMC3, TRAF5, SELL, CCL19, CCAR1, ARL13B, SAMSN1, PIK3AP1, DNAJB4, APOBEC3A, HPGDS, FCGR3A, ANP32A, CHIT1, CARD16, P2RY14, CTR9, DYRK3, MPHOSPH10, SH3PXD2B, GLT8D1, FAHD2A, GBP1P1, EVI2B, CWC22, MPLKIP, PI4K2B, DCAF13, IRF2, LUC7L3, TMEM133, SYAP1, ACAD8, PLCG1, ZC3H7A, POU2AF1, RTN3, HMGN3, PPIG, PLAGL1, ILK, SMAD7, FAM26F, HNRNPC, MCTS1, CAPZA1, POLR2K, GIMAP7, C1D, CYP51A1, ITM2A, GBP3, CBY1, DENND4C, SREK1, FCRLA
Airway	KCNK3, LOC101927769, CPNE4, VGLL3, AQP2, NMNAT2, IFITM10, AHRR, JPH2, PSD2, CDH11, DCTN1AS1, FGF22, SMIM1, SYNPO2L, LOC101927914, ELFN2, TAL1, FRMD8P1, TSPAN18, CLEC5A, GRP, JAKMIP3, LOC102546299, SLC30A3, PLK5, LCN8, GBX1, LINC00269, ITLN1, KCNIP3, EWSAT1, PITX2, TPH1, CDH6, PRICKLE2AS3, SULT4A1, SOX9AS1, C1QTNF4, SEMA5B, FRMD1, KCNJ4, CLEC14A, NAT16, KCNQ2, LINC00942, CBLN4, LOC101927870, GLB1L3, PITX3, PSMA8, NR1I2, ARHGEF10, ELAVL3, LOC400622, KCNA1, NKD1, SCUBE3, LOC101929552, MAPK12, OBP2A, RPL13AP17, OR5K1, NHLH2, PAX1, TCF4AS1, SGK2, PTGIR, GFRA2, COL8A1, GREM2, LINC00652, UNC5C, GPBAR1, LOC254028, VWC2, HHLA1, MYOZ3, KIZAS1, ABCB6, DKKL1, ATP8B5P, ADAM11, FAM167AAS1, HAP1, SYT16, PIK3CDAS1, PHACTR3, LOC158434, HIF3A, OR5H1, BDNF, CALCA, APLP1, ZIC1, LRRN4, FBXO17, BMP4, KLC3, MEIS3, NTRK3, SYT1, MIR924HG, DDN, AVPR1A, C10orf126, BRSK2, LOC101927636, LHX6, CYP1B1AS1, INMT, CTD2350J17.1, ART3, LINC01056, C1orf127, RAMP2, ATOH7, LHX9, CNPY1, DHRS2
Blood	CCL3L1, FCER1A, TRIM6

Finally, we cross-processed the overlapping factors that were strictly screened for the two diseases and finally got 42 common host factors.

### TF-Gene Interaction and Gene-miRNA Interaction

**FIGURE 2:**
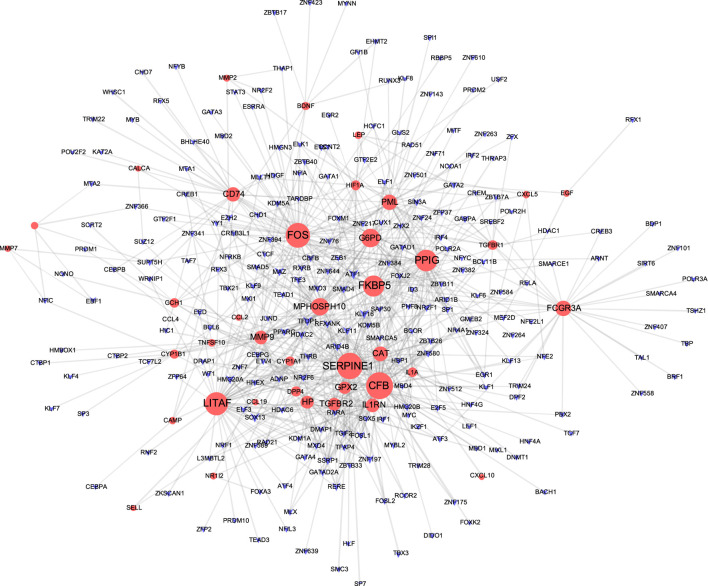
Network of TF gene interpaly with shared targets. The red node is the shared target, and the purple node shows TF-gene.

**FIGURE 3:**
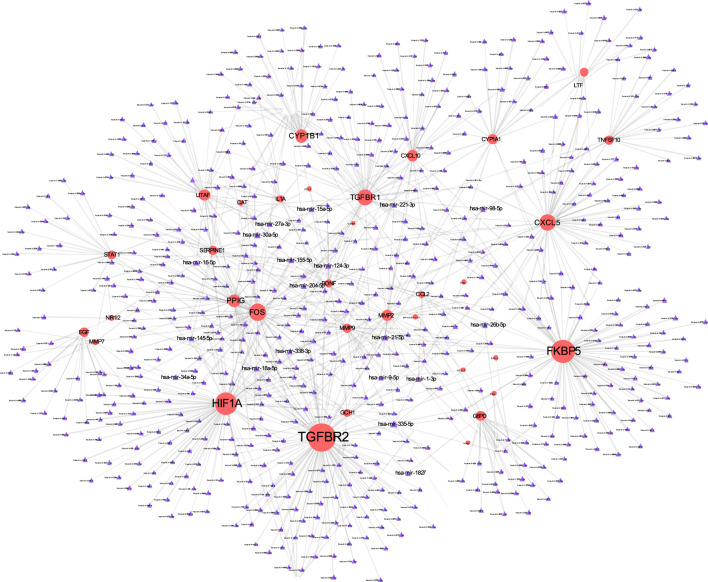
Gene-miRNA interaction network. The red node represents the shared target, and the purple node shows miRNA.

**FIGURE 4:**
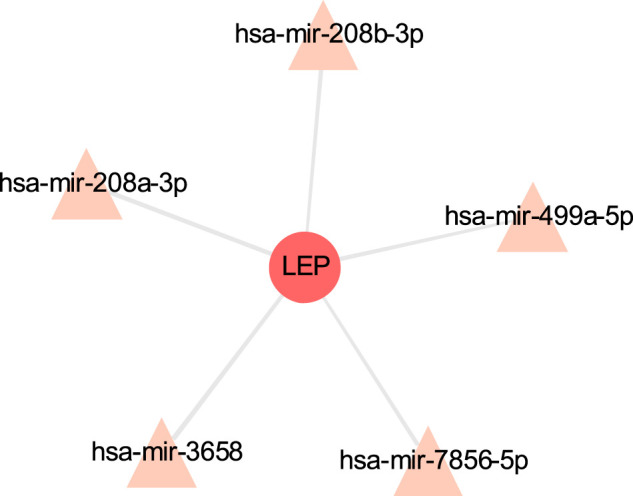
Gene-miRNA interaction sub-network. The red node is the shared target, and the pink node shows miRNA.

The TF-gene interaction network consists of 285 nodes and 717 edges (Figure 2 and Supplementary Table S2). Among them, CFB is regulated by 58 TF-genes, FOS is regulated by 51 genes, and FKBP5 is regulated by 43 TF-genes. See Supplementary Table S2 for details. In addition, the gene-miRNA interaction network has a subnet with at least 3 nodes as shown below. Subnet 1 (Figure 3 and Supplementary Table S3) is composed of 638 nodes and 879 edges, and subnet 2 (Figure 4 and Supplementary Table S3) is composed of 6 nodes and 5 edges.

### Protein-Protein Interaction Network and MCODE Analysis

**FIGURE 5:**
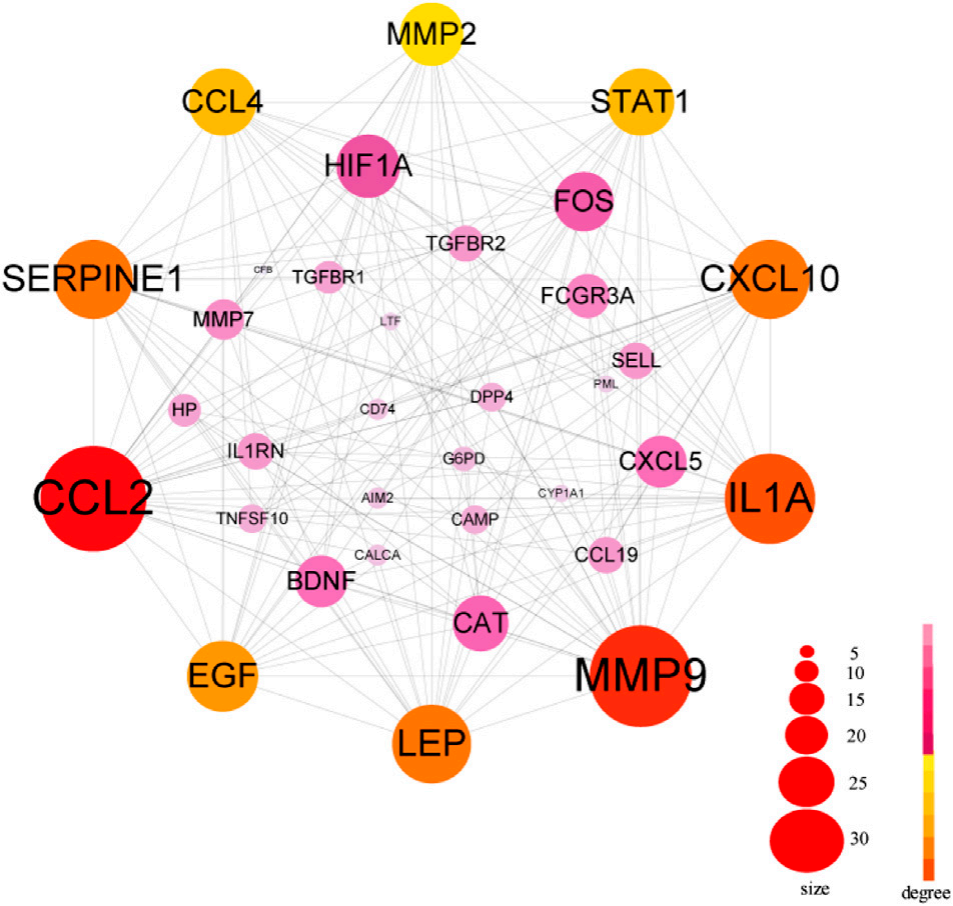
PPI network of common host factors for COVID-19 and COPD. In this figure, the circled nodes represent host factors, and the edges represent the interactions between nodes. The larger the circle, the darker the color, the higher the importance, the thicker the line, the greater the interaction. The top ten genes with degree value are highlighted in another color, and other genes are shown in pink.

**FIGURE 6:**
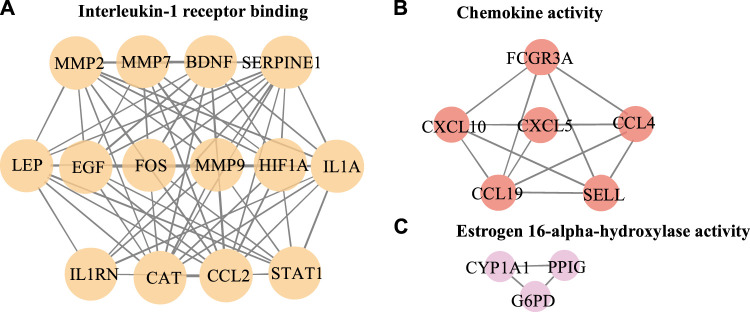
Further MCODE analysis based on PPI network. The nodes circled in the figure represent the host factor, and the edges represent the interaction between the nodes. Different colors represent different modules.

The PPI network in this study was generated by string based on 42 common host genes and then introduced into Cytoscape for visual representation and network topology analysis. In the end, we get 42 nodes, 199 edges, and the average node degree is 9.48. In this study, we rank the nodes in the PPI network according to their degree values. The top ten targets are CCL2, MMP9, IL1A, LEP, SERPINE1, CXCL10, EGF, CCL4, STAT1, and HIF1A (Figure 5). In addition, set the cluster finding parameters (node score cutoff: 0.2, k-core: 2, max depth: 100), through MCODE analysis (Figure 6), we classify 42 host factors, and finally get three different biological functions subnet. Module A mainly reflects the interleukin-1 receptor binding function. In its visualization diagram, we can clearly see that MMP9, IL1A, FOS, LEP, EGF, HIF1A and other nodes occupy important positions. Module B mainly functions as the Chemokine activity. Among them, the degree of CXCL10, CXCL5, and CCL4 is higher. In module C, the active function of estrogen 16-alpha-hydroxylase is more prominent. The analysis of its function shows that the signal receptor binding function mediated by CCL2 and MMP9 is the potential mechanism of the host factor interaction network between COVID-19 and COPD.

### GO, KEGG Enrichment Analysis

**FIGURE 7:**
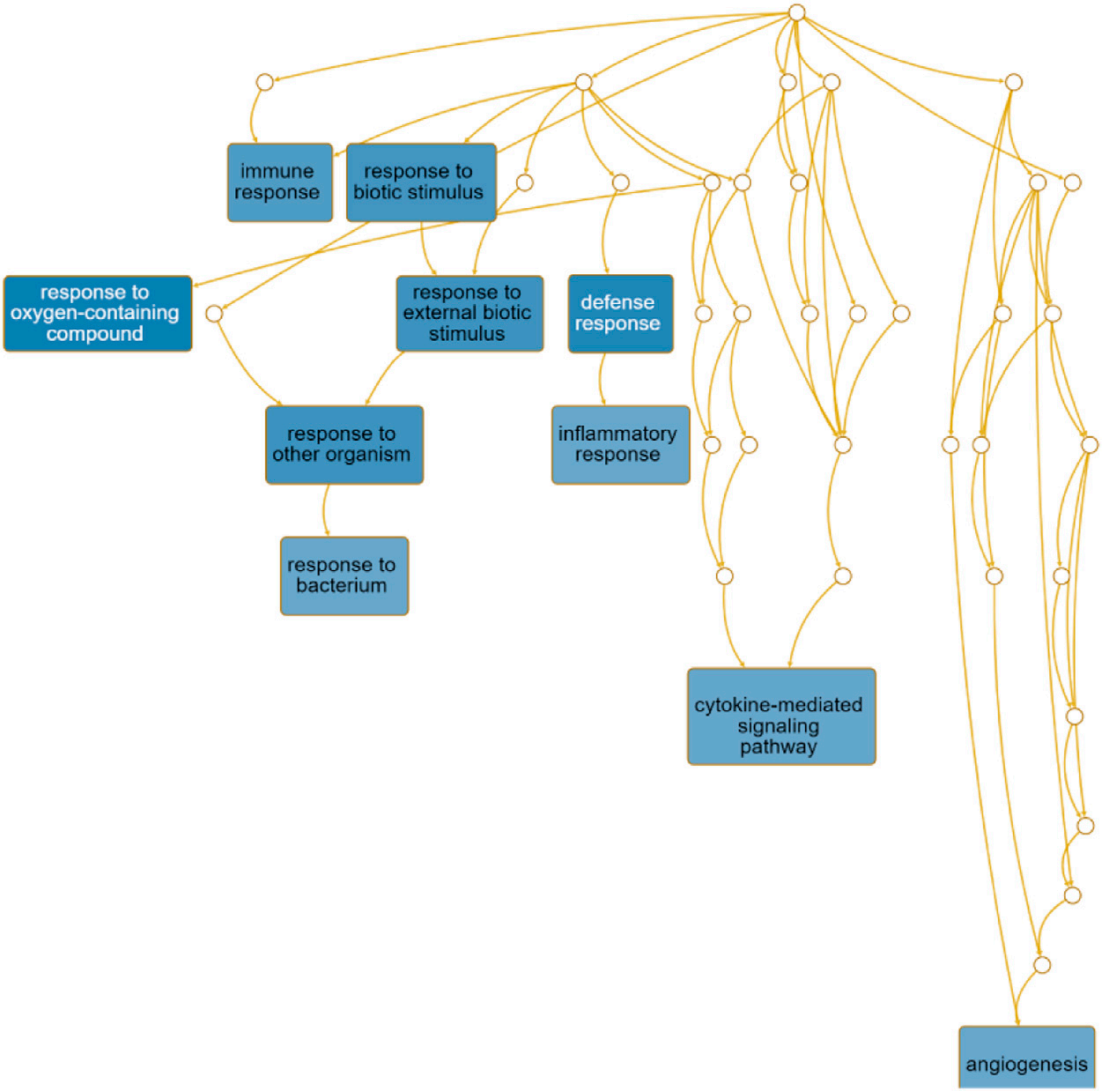
GO biological process analysis. DAG maps the causal relationship between the arrows between the variables and the nodes. The absence of arrows between nodes means that there is no causality and precedence, and the nodes can be measured or cannot be measured. The node whose position is in the front is the parent node, and the one in the back is the child node.

**FIGURE 8:**
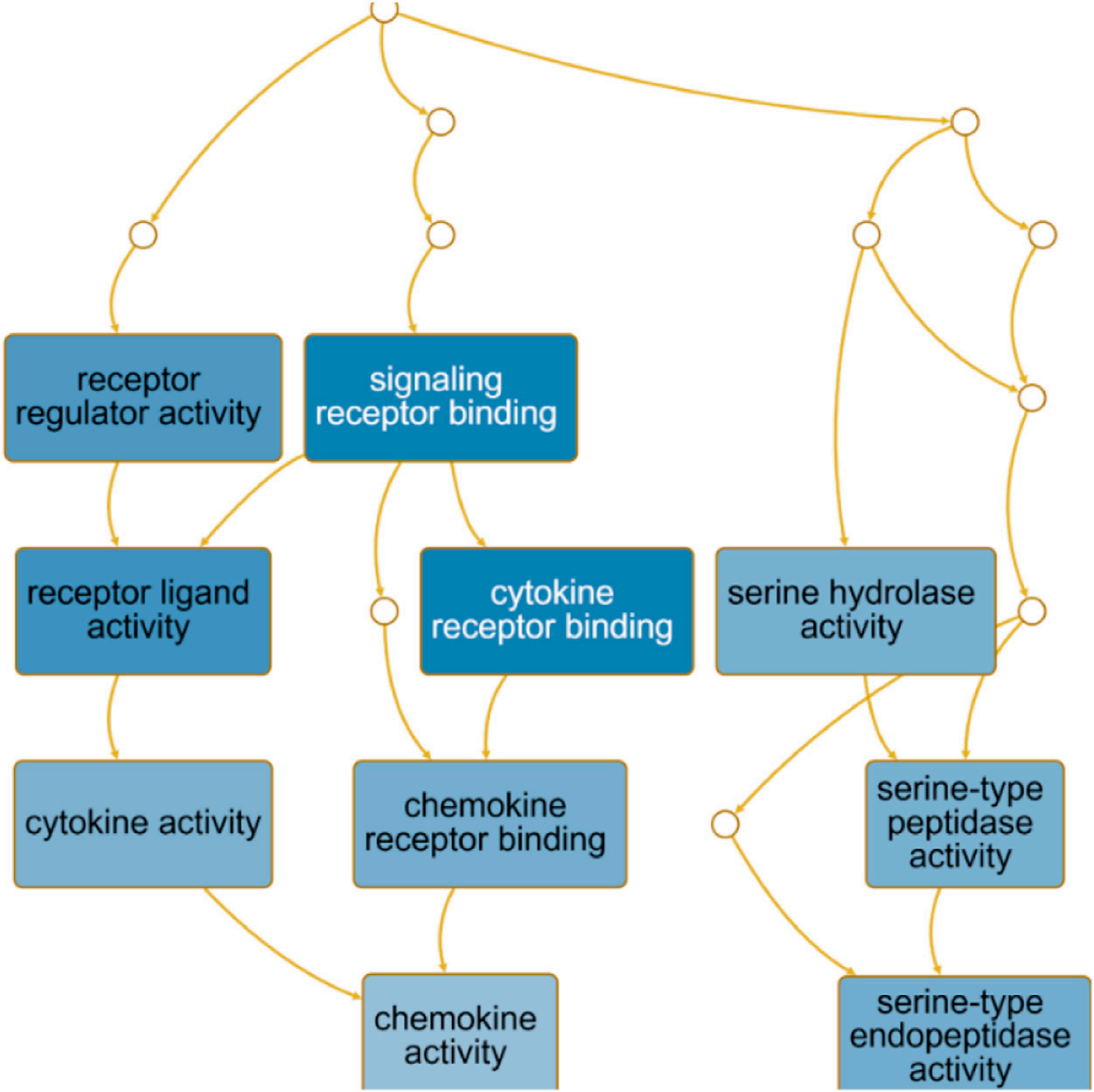
GO molecular function analysis. DAG maps the causal relationship between the arrows between the variables and the nodes. The absence of arrows between nodes means that there is no causality and precedence, and the nodes can be measured or cannot be measured. The node whose position is in the front is the parent node, and the one in the back is the child node.

The directed acyclic graph (DAG) analysis of GO’s biological process shows that the biological processes (Figure 7 and Table 3) in COVID-19 and COPD comorbidities mainly have 5 branches, of which angiogenesis, cytokine-mediated signaling pathway, and cell migration are separate Branch. The defense response is immediately followed by the inflammatory response. At the same time, immune response and response to biotic stimulus are carried out in parallel, and response to biotic stimulus links response to external biotic stimulus and response to oxygen-containing compound processes, and finally ends with response to other organism. The molecular functions in COVID-19 and COPD comorbidities (Figure 8 and Table 4) mainly have two major branches, among which the enrichment ratio of chemokine activity is the highest. Receptor regulator activity, receptor ligand activity, cytokine activity and chemokine activity belong to the same branch; signaling receptor binding, cytokine receptor binding, chemokine receptor binding and chemokine activity belong to the same branch; serine hydrolase activity, serine-type peptidase activity and serine-type endopeptidase activity both belong to another branch.

**TABLE 3:** GO-BP enrichment analysis.

GeneSet	Description	Size	Overlap	Expect	Enrichment Ratio	*p* Value	FDR
GO:1901700	response to oxygen-containing compound	1,556	24	3.82837254	6.268982	5.11E-15	4.64E-11
GO:0006952	defense response	1,518	23	3.73487758	6.158167	4.02E-14	1.83E-10
GO:0051707	response to other organism	897	18	2.206973116	8.155967	7.61E-13	1.80E-09
GO:0043207	response to external biotic stimulus	899	18	2.211893903	8.137823	7.90E-13	1.80E-09
GO:0009607	response to biotic stimulus	926	18	2.278324532	7.900543	1.30E-12	2.37E-09
GO:0006955	immune response	1919	23	4.721495439	4.871338	5.78E-12	8.76E-09
GO:0019221	cytokine-mediated signaling pathway	705	15	1.734577532	8.647639	4.89E-11	6.35E-08
GO:0006954	inflammatory response	717	15	1.764102256	8.502908	6.20E-11	6.88E-08
GO:0009617	response to bacterium	595	14	1.463934229	9.563271	6.81E-11	6.88E-08
GO:0001525	angiogenesis	487	13	1.198211714	10.8495	8.08E-11	7.30E-08

**TABLE 4:** GO-MF enrichment analysis.

GeneSet	Description	Size	Overlap	Expect	Enrichment Ratio	*p* Value	FDR
GO:0005102	signaling receptor binding	1,538	19	3.783177	5.022233	6.55E-10	8.21E-07
GO:0005126	cytokine receptor binding	274	10	0.673986	14.8371	8.75E-10	8.21E-07
GO:0048018	receptor ligand activity	468	11	1.151188	9.555347	1.12E-08	7.03E-06
GO:0030545	receptor regulator activity	514	11	1.264339	8.700199	2.95E-08	1.38E-05
GO:0004252	serine-type endopeptidase activity	182	7	0.447684	15.63602	2.71E-07	1.02E-04
GO:0042379	chemokine receptor binding	61	5	0.150048	33.32267	3.76E-07	1.18E-04
GO:0008236	serine-type peptidase activity	204	7	0.5018	13.94978	5.86E-07	1.57E-04
GO:0017171	serine hydrolase activity	208	7	0.511639	13.68152	6.68E-07	1.57E-04
GO:0005125	cytokine activity	217	7	0.533777	13.11408	8.88E-07	1.85E-04
GO:0008009	chemokine activity	47	4	0.115611	34.59886	5.21E-06	9.70E-04

At the same time, pathway enrichment analysis showed (Table 5) that cytokine-cytokine receptor interaction, AGE-RAGE signaling pathway in diabetic complications, viral protein interaction with cytokine and cytokine receptor, osteoclast differentiation and IL-17 signaling pathway play an important role between COVID and COPD.

**TABLE 5:** KEGG enrichment analysis.

ID	Description	GeneRatio	*p* value	p.adjust	Qvalue	Count
hsa04060	Cytokine-cytokine receptor interaction	11	6.17E-08	9.44E-06	6.37E-06	11
hsa04933	AGE-RAGE signaling pathway in diabetic complications	7	3.25E-07	2.49E-05	1.68E-05	7
hsa04061	Viral protein interaction with cytokine and cytokine receptor	6	6.08E-06	0.00031	0.000209	6
hsa04380	Osteoclast differentiation	6	2.52E-05	0.000963	0.000649	6
hsa04657	IL-17 signaling pathway	5	6.99E-05	0.00214	0.001443	5
hsa05142	Chagas disease	5	0.000103	0.002589	0.001746	5
hsa05164	Influenza A	6	0.000132	0.002589	0.001746	6
hsa04659	Th17 cell differentiation	5	0.000135	0.002589	0.001746	5
hsa04668	TNF signaling pathway	5	0.000161	0.002733	0.001842	5
hsa04062	Chemokine signaling pathway	6	0.00024	0.00367	0.002474	6
hsa04926	Relaxin signaling pathway	5	0.000311	0.004262	0.002874	5
hsa04068	FoxO signaling pathway	5	0.000334	0.004262	0.002874	5
hsa05212	Pancreatic cancer	4	0.000414	0.004483	0.003022	4
hsa05140	Leishmaniasis	4	0.000435	0.004483	0.003022	4
hsa05418	Fluid shear stress and atherosclerosis	5	0.000439	0.004483	0.003022	5
hsa05208	Chemical carcinogenesis - reactive oxygen species	6	0.000536	0.005122	0.003453	6
hsa05210	Colorectal cancer	4	0.000662	0.005962	0.00402	4
hsa05235	PD-L1 expression and PD-1 checkpoint pathway in cancer	4	0.000754	0.00641	0.004322	4
hsa05161	Hepatitis B	5	0.000883	0.006531	0.004403	5
hsa05323	Rheumatoid arthritis	4	0.00089	0.006531	0.004403	4
hsa05219	Bladder cancer	3	0.000896	0.006531	0.004403	3
hsa00380	Tryptophan metabolism	3	0.000962	0.006692	0.004512	3
hsa04620	Toll-like receptor signaling pathway	4	0.00135	0.008984	0.006057	4
hsa05152	Tuberculosis	5	0.001416	0.009024	0.006084	5
hsa04010	MAPK signaling pathway	6	0.002243	0.01373	0.009257	6

### Tissue Specific Enrichment Analysis of Host Factor Interaction Network

**FIGURE 9:**
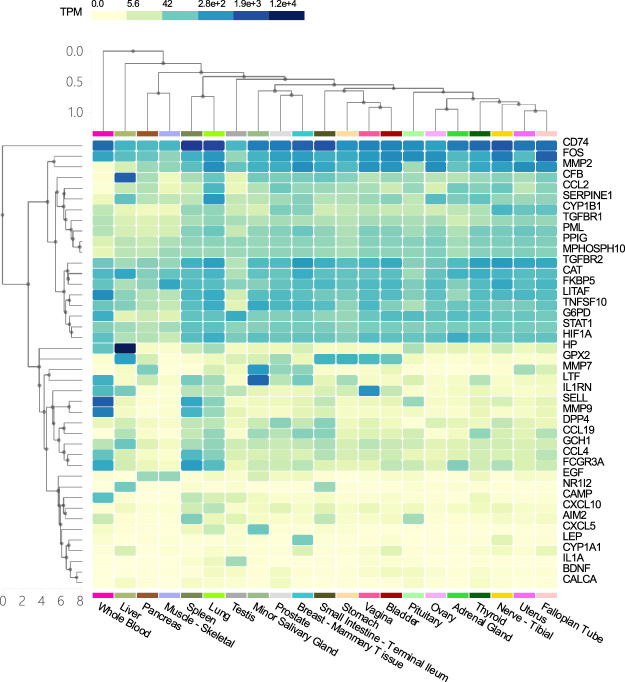
Tissue specific enrichment analysis graph. The horizontal axis in the figure represents different tissues, and the vertical axis represents the corresponding distribution of host factors in the tissues. The darker the vertical axis, the higher the specific distribution density of the host factor in the corresponding tissue.

Tissue specific enrichment analysis showed that the co-host factors of COVID-19 and COPD comorbidities were most densely distributed in the lungs, spleen, liver, blood, small salivary glands, breast-breast tissue, prostate and vagina (Figure 9).

### Drug Prediction Through Common Host Factors

Based on a series of bioinformatics explorations on the interaction network between COVID-19 and COPD host factors, we finally made predictions about possible effective intervention drugs. Our research found that dexamethasone, estradiol, progesterone, and nitric oxide have certain intervention effects, as shown in Table 6.

**TABLE 6:** Drug stitch enrichment analysis.

Enrichment FDR	Genes in list	Total genes	Functional category
1.02E-25	23	409	STITCH dexamethasone (CID000005743)
1.02E-25	22	340	STITCH dexamethasone (CID100003003)
2.36E-18	18	367	STITCH estradiol (CID100000450)
4.94E-18	17	310	STITCH progesterone (CID000005994)
3.30E-16	14	194	STITCH nitric oxide (CID100000945)

## Discussion

All the work done in this research is to explore possible interaction pathways between COVID-19 and COPD from the perspective of host factors. On the basis of the front, we tried to find supporting evidence for the increased risk of pneumonia and poor prognosis when COPD patients were simultaneously infected with the SARS-COV-2 virus, and finally made reasonable predictions about the drug components that may be effective for intervention. The first step of our work was to screen out appropriate disease datasets/databases, find out the potential host factor of COVID-19/COPD, and then count the overlapping genes to obtain 42 common host factors. Based on the common host factors, a series of bioinformatics analyses were carried out.

### Intersection Genes of COVID-19 and COPD Show the Key Host Factors of Comorbidity

After rigorously screening and processing genes, we have identified multiple shared genes exposed in the immune response of COVID-19 with COPD conditions (including CCL2, MMP9, IL1A, LEP, SERPINE1, CXCL10, EGF, CCL4, STAT1, and HIF1A). Most of these common genes have been shown to be related to the strong biological relevance of pathogenesis and pathology of COVID-19 and COPD.

### TF-Gene Interaction and Gene-miRNA Interaction Analysis

Transcriptome analysis of host cells after virus infection is helpful to identify the dynamics of host immune response, so it is necessary to understand the expression of host factors after co-infection of COVID-19 and COPD. The TF gene acts as a regulator based on genetic expression. In this study, we found that CFB, FOS and FKBP5 showed a high degree of interaction with other TF genes. CFB is one of the highly induced supplemental genes in response to SARS-CoV-2. The cell penetration inhibitor of CFB may block the active supplement C3a of respiratory epithelial cells produced by SARS-CoV-2 infection (Yan et al., 2021). Not only that, our research shows that the hypoxia-inducible factor HIF1A occupies an important position in the gene-miRNA network. Previous studies have also shown that HIF dysfunction is closely related to inflammatory airway conditions, and HIF1A plays a central role in the development of COPD and other lung diseases (Kelchtermans et al., 2021).

### PPI and MCODE Analysis Reveal Essential Host Genes and Distinct Biological Functions for Comorbidity

Chemokines are small molecules (8–12 kDa) of a large family of cytokines associated with various biological functions, and several studies have now established the critical role of chemokines in the development and progression of chronic obstructive pulmonary disease (COPD). Cigarette smoke or other irritants can activate alveolar macrophages and airway epithelial cells, releasing chemokines that attract circulating leukocytes to the lungs. In addition, various factors such as air pollution can induce the release of chemokines from resident cells by triggering the release of damage-associated molecular patterns (DAMP) that bind to specific pattern recognition receptors (PRR) (Ko et al., 2016). Chemokines (CCL2, CCL4) also occupy an important position in this study. In the lung, CCL2 is mainly produced by lung macrophages, T cells and endothelial cells and is involved in endothelial and pulmonary epithelial cell proliferation, migration and wound closure, and is associated with a variety of diseases with disorders of lung inflammation, including COPD (Henrot et al., 2019), acute respiratory distress syndrome, allergic asthma and idiopathic pulmonary fibrosis (Rose et al., 2003). In contrast, for patients with COVID-19, the elevated cytokines in the blood lie in the over-induction of the cytokine storm. In addition, the interaction between SARS-CoV spiking protein and ACE2 receptor mediated the phosphorylation of ERK1/2, which also led to the upregulation of CCL2 expression (Chen et al., 2010). In the early stages of SARS-CoV-2 infection, chemokine (CCL2) is broadly up-regulated by pro-inflammatory cytokine stimulation as a chemoattractant for effector cells such as monocytes, neutrophils, and leukocytes from the blood to the site of tissue injury. In addition, CCL2 can act as an autocrine factor that promotes viral replication in infected macrophages (Sabbatucci et al., 2015). In a high-density antibody microarray study of serum proteins from COVID-19 patients, a significant correlation between CCL2 and CXCL10-mediated cytokine signaling pathways has been demonstrated (Hou et al., 2020). This study suggests that CCL2 and CXCL10 have the potential to be used as anti-inflammatory targets for COVID-19 therapy (Zhang et al., 2020).

Matrix metalloproteinase 9 (MMP9) is particularly associated with COPD pathophysiology characterized by tissue remodeling. MMP9 mediates pulmonary inflammation through neutrophil chemotaxis, extracellular matrix degradation and enhanced inflammation, which is a key feature of the acute exacerbation phase of COPD (Mercer et al., 2005; Wells et al., 2015). Earlier reports suggested that human coronavirus infection increases MMP9 secretion (Desforges et al., 2007). Similarly, recent studies suggest that MMP9 stimulates the migration of inflammatory cells and further exacerbates lung tissue destruction by promoting inflammation and degradation of the pulmonary capillary barrier (Davey et al., 2011), which may serve as one of the early indicators of respiratory failure in patients with COVID-19 (Ueland et al., 2020).

The MCODE analysis unearthed modules with potentially different biological functions in the network nodes, providing a clearer direction for bioinformatics analysis. In this study, 3 subnetworks were obtained after dividing the 42 obtained host factors for COVID-19 and COPD comorbidity. Among them, module A focused on receptor binding, including interleukin-1 receptor binding, the RNA polymerase II core promoter sequence-specific DNA binding, the histone acetyltransferase binding, metalloendopeptidase activity, growth factor receptor binding, cytokine receptor binding, receptor ligand activity, receptor regulator activity and signaling receptor binding, etc. CCL2, MMP9, IL1A, HIF1A, and LEP occupy a prominent position in the whole module. Accumulating evidence suggests that infection with viruses activates extracellular signaling and induces IL-1 production (Liu et al., 2013). IL1A is upregulated in patients with mild COVID-19 and also enriched in alveolar lavage fluid of severe patients, playing an important role in innate immune virus infection (Shaath et al., 2020) and IL1A, as a pro-inflammatory cytokine, plays an important role in smoke-induced neutrophilic inflammation, dendritic cell recruitment and activation in CODP patients also plays a central role (Botelho et al., 2011).

### GO, KEGG Highlights the Immune Mechanisms of Host Factors and Significant Shared Signaling Pathways

The directed acyclic graph (DAG), as a visual representation of the causal hypothesis (Suttorp et al., 2015), has clear advantages in estimating the effect of one variable on another and is a common tool for determining appropriate adjustment strategies for epidemiological analyses (Ferguson et al., 2020). This structured approach facilitates visual clarification of the underlying relationships and serves as a visual aid in scientific discussions. Therefore, the GO analysis in this study abandoned the traditional network diagram format and adopted a DAG visualization approach to elucidate the mechanisms of comorbidity between COVID-19 and COPD.

The importance of angiogenesis is underscored by its separate branch in the biological processes of comorbidity, which is also the highest enrichment ratio of all genes. A 7-person clinical trial (Ackermann et al., 2020) had reported severe SARS-CoV-2 virus-associated endothelial damage and extensive vascular thrombosis in the lung cells of Covid-19 patients due to excessive cytokine storm, and significant neointimal growth in the lungs of Covid-19 patients through an infected angiogenic mechanism (Magro et al., 2020). In contrast, COPD, a pulmonary and systemic inflammatory process with progressive obstruction of pulmonary airflow, epithelial-mesenchymal transition and extracellular matrix remodeling similarly affects pulmonary and airway angiogenesis (Eapen et al., 2018). Our findings suggest that chemokine activity also plays an important role in the biological function of comorbidities. Chemokines recruit innate and adaptive immune cells to sites of inflammation, enhance their cytotoxic function and inhibit viral host responses, limiting viral infection (Melchjorsen et al., 2003). At the same time, viruses link innate and adaptive immune responses by inducing the production of inflammatory chemokines and promoting Th1-polarized immune responses. For COVID-19, CCL2 recruits neutrophils, monocytes, and macrophages, and CXCL9 and CXCL16 recruit T cells and NK cells to the site of viral infection (Proudfoot, 2002; Xu et al., 2020). Interestingly, CXCL10 increases with disease severity and is a key marker for detection in asymptomatic infected individuals (Chi et al., 2020). And chemoreceptors have long been a fertile area for research as anti-inflammatory therapeutic targets in COPD (Donnelly and Barnes, 2006).

In addition, KEGG enrichment analysis revealed important shared signaling pathways, cytokine-cytokine receptor interactions, viral protein interactions with cytokines and cytokine receptors, and IL-17 signaling pathways in diabetic complications. The No. 1 cytokine-cytokine receptor interaction pathway is enriched with 11 host factors, mainly chemokine ligands and transforming growth factor β receptors. Viral infection and inflammation will cause changes in TGF-β activity (Xia et al., 2017).

### Tissue Specific Enrichment Analysis Indicates the Expression of Certain Specific Host Factors

Our results suggest that the common host factors for COVID-19 and COPD comorbidity are most densely distributed in the lung, spleen, liver, blood, minor salivary glands, breast tissue, prostate, and vagina. As to why genes selected from alveolar lavage fluid, lung tissue, airway and blood samples are enriched in other organs and tissues, we think it may be due to the flow of blood that leads to the linkage between different tissues, or the intersection genes may also be derived from other tissues at the same time, because the genes from the database are not limited to samples of blood, lung tissue, alveolar lavage fluid and airway.

### The Intervention Drug Reveals Therapeutic Implications for COPD Patients With COVID-19

As shown in the potential intervention drugs or ingredients for COVID-19 and COPD comorbidities (Table 6), our study found that dexamethasone, estradiol, progesterone, and nitric oxide, etc. all demonstrated effective intervention. In a randomized controlled trial, the RECOVERY Collaborative Group (Horby et al., 2021) found that using dexamethasone at a daily dose of 6 mg for 10 consecutive days reduced mortality for 28 days in patients receiving respiratory support for COVID-19. Glucocorticoids are also recommended in the updated guidelines of the United Kingdom chief medical officers and the National Institutes of Health in the United States for inpatient use of COVID-19. There is no definitive clinical data on the clinical outcomes of the use of glucocorticoids in COPD patients who are infected with COVID-19 at the same time (Halpin et al., 2020; Halpin et al., 2021), but our results suggest that there is bioinformatics evidence for the use of dexamethasone for treatment. Nevertheless, more laboratory and clinical trials are needed before dexamethasone becomes a potential therapeutic drug in the future. In addition, studies have found that 17β-estradiol administration can effectively reduce the up-regulation of ACE2-dependent NOX2, MCP-1 and ROS, and alleviate endothelial dysfunction and multiple organ failure mediated by COVID-19 inflammation during the pathogenesis (Youn et al., 2021). Experiments have pointed out that the combination of progesterone and glucocorticoids can synergistically reduce lung inflammation in mice caused by chronic ozone exposure (Fei et al., 2017). And, progesterone has a certain role in COPD airway remodeling (Zhang et al., 2018). In addition, estrogen can also promote the separation of endothelial nitric oxide synthase (eNOS) from plasma membrane acupoints, thereby activating NO pathways and vascular adsorption, and playing a role in regulating blood vessels (Hisamoto and Bender, 2005). These data provide support for our research results, but there are no relevant clinical and experimental studies on the use of this ingredient in COVID-19 and COPD comorbidities. This will be one of the contents of our future work research.

## Conclusion

In order to explore the mechanism of co-morbidity between COVID-19 and COPD, after carefully screening the COVID-19 and COPD data sets and strictly processing co-host genes, we conducted a series of bioinformatics analyses from the perspective of host factor interactions, and initially discovered drugs or active ingredients for potential interventions. We found that the main biological process of COPD patients infected with COVID-19 is angiogenesis, and the main molecular functionis chemokine activity. In addition, we also found that the cytokine-cytokine receptor interactions signaling pathway is a common pathway for the progression of the two diseases. Finally, we concluded that dexamethasone, estradiol, progesterone, and nitric oxide are potentially effective therapeutic drugs, providing a clearer direction for future clinical research.

## Limitation

First of all, our research is based on co-expressed genes, involving non-coding RNA, but we have not conducted studies on post-translational modification and interference with other metabolites. This is related to the content of our research, but they are not the subject of this research. Therefore, we will supplement the research in future work. Secondly, although the study selected sample data from airway, lung, and peripheral blood for tissue-specific enrichment analysis, the results showed that the co-host factors of COVID-19 and COPD comorbidities were also enriched in spleen, liver, blood, minor salivary glands, breast tissue, prostate, and vagina. We speculate that the peripheral blood may mediate this process, or it may be because we also selected genes from the database. The genes in the database are not distinguished according to the source of the tissue, so there is a certain amount of confounding. Finally, given the limitations of bioinformatics predictions, candidate drugs may also affect counter-regulatory genes not identified in this study. We must admit that this is the limitation of our research. Therefore, we are trying to find positive intermediary evidence to support the prediction results, and we also look forward to future *in vivo* and *in vitro* experiments to prove this.

## Data Availability

The datasets presented in this study can be found in online repositories. The names of the repository/repositories and accession number(s) can be found in the article/Supplementary Material.

## References

[B1] Ackermann M. Verleden S. E. Kuehnel M. Haverich A. Welte T. Laenger F. (2020). Pulmonary Vascular Endothelialitis, Thrombosis, and Angiogenesis in Covid-19. N. Engl. J. Med. 383 (2), 120–128. 10.1056/NEJMoa2015432 32437596 PMC7412750

[B2] Bafadhel M. McKenna S. Terry S. Mistry V. Reid C. Haldar P. (2011). Acute Exacerbations of Chronic Obstructive Pulmonary Disease: Identification of Biologic Clusters and Their Biomarkers. Am. J. Respir. Crit. Care Med. 184 (6), 662–671. 10.1164/rccm.201104-0597OC 21680942

[B83] Blanco-Melo D. Nilsson-Payant B. E. Liu W. C. Uhl S. Hoagland D. Møller R.(2020). Imbalanced Host Response to SARS-CoV-2 Drives Development of COVID-19. Cell 181 (5), 1036–1045.e9. 10.1016/j.cell.2020.04.026 32416070 PMC7227586

[B3] Botelho F. M. Bauer C. M. Finch D. Nikota J. K. Zavitz C. C. Kelly A. (2011). IL-1α/IL-1R1 Expression in Chronic Obstructive Pulmonary Disease and Mechanistic Relevance to Smoke-Induced Neutrophilia in Mice. PLoS One 6 (12), e28457. 10.1371/journal.pone.0028457 22163019 PMC3232226

[B4] Cai Q. Chen J. Xu L. (2020). Response to Comment on Cai et al. Obesity and COVID-19 Severity in a Designated Hospital in Shenzhen, China. Diabetes Care 2020;43:1392-1398. Diabetes Care 43 (7), e162–1398. 10.2337/dci20-0034 32958625

[B5] Cecconi M. Piovani D. Brunetta E. Aghemo A. Greco M. Ciccarelli M. (2020). Early Predictors of Clinical Deterioration in a Cohort of 239 Patients Hospitalized for Covid-19 Infection in Lombardy, Italy. J. Clin. Med. 9 (5), 1548. 10.3390/jcm9051548 32443899 PMC7290833

[B6] Chen I. Y. Chang S. C. Wu H. Y. Yu T. C. Wei W. C. Lin S. (2010). Upregulation of the Chemokine (C-C Motif) Ligand 2 via a Severe Acute Respiratory Syndrome Coronavirus Spike-ACE2 Signaling Pathway. J. Virol. 84 (15), 7703–7712. 10.1128/JVI.02560-09 20484496 PMC2897593

[B7] Chi Y. Ge Y. Wu B. Zhang W. Wu T. Wen T. (2020). Serum Cytokine and Chemokine Profile in Relation to the Severity of Coronavirus Disease 2019 in China. J. Infect. Dis. 222 (5), 746–754. 10.1093/infdis/jiaa363 32563194 PMC7337752

[B8] Daniloski Z. Jordan T. X. Wessels H. H. Hoagland D. A. Kasela S. Legut M. (2021). Identification of Required Host Factors for SARS-CoV-2 Infection in Human Cells. Cell 184 (1), 92–e16. 10.1016/j.cell.2020.10.030 33147445 PMC7584921

[B9] Davey A. McAuley D. F. O'Kane C. M. (2011). Matrix Metalloproteinases in Acute Lung Injury: Mediators of Injury and Drivers of Repair. Eur. Respir. J. 38 (4), 959–970. 10.1183/09031936.00032111 21565917

[B10] Desforges M. Miletti T. C. Gagnon M. Talbot P. J. (2007). Activation of Human Monocytes after Infection by Human Coronavirus 229E. Virus. Res. 130 (1-2), 228–240. 10.1016/j.virusres.2007.06.016 17669539 PMC7114174

[B11] Donnelly L. E. Barnes P. J. (2006). Chemokine Receptors as Therapeutic Targets in Chronic Obstructive Pulmonary Disease. Trends Pharmacol. Sci. 27 (10), 546–553. 10.1016/j.tips.2006.08.001 16911834

[B12] Eapen M. S. Hansbro P. M. Larsson-Callerfelt A. K. Jolly M. K. Myers S. Sharma P. (2018). Chronic Obstructive Pulmonary Disease and Lung Cancer: Underlying Pathophysiology and New Therapeutic Modalities. Drugs 78 (16), 1717–1740. 10.1007/s40265-018-1001-8 30392114

[B13] Fei X. Bao W. Zhang P. Zhang X. Zhang G. Zhang Y. (2017). Inhalation of Progesterone Inhibits Chronic Airway Inflammation of Mice Exposed to Ozone. Mol. Immunol. 85, 174–184. 10.1016/j.molimm.2017.02.006 28279894

[B14] Feng Y. Ling Y. Bai T. Xie Y. Huang J. Li J. (2020). COVID-19 with Different Severities: A Multicenter Study of Clinical Features. Am. J. Respir. Crit. Care Med. 201 (11), 1380–1388. 10.1164/rccm.202002-0445OC 32275452 PMC7258639

[B15] Ferguson K. D. McCann M. Katikireddi S. V. Thomson H. Green M. J. Smith D. J. (2020). Evidence Synthesis for Constructing Directed Acyclic Graphs (ESC-DAGs): a Novel and Systematic Method for Building Directed Acyclic Graphs. Int. J. Epidemiol. 49 (1), 322–329. 10.1093/ije/dyz150 31325312 PMC7124493

[B16] Garg S. Kim L. Whitaker M. O'Halloran A. Cummings C. Holstein R. (2020). Hospitalization Rates and Characteristics of Patients Hospitalized with Laboratory-Confirmed Coronavirus Disease 2019 - COVID-NET, 14 States, March 1-30, 2020. MMWR Morb Mortal Wkly Rep. 69 (15), 458–464. 10.15585/mmwr.mm6915e3 32298251 PMC7755063

[B17] Ge S. X. Jung D. Yao R. (2020). ShinyGO: a Graphical Gene-Set Enrichment Tool for Animals and Plants. Bioinformatics 36 (8), 2628–2629. 10.1093/bioinformatics/btz931 31882993 PMC7178415

[B18] George S. N. Garcha D. S. Mackay A. J. Patel A. R. Singh R. Sapsford R. J. (2014). Human Rhinovirus Infection during Naturally Occurring COPD Exacerbations. Eur. Respir. J. 44 (1), 87–96. 10.1183/09031936.00223113 24627537

[B19] Goyal P. Choi J. J. Pinheiro L. C. Schenck E. J. Chen R. Jabri A. (2020). Clinical Characteristics of Covid-19 in New York City. N. Engl. J. Med. 382 (24), 2372–2374. 10.1056/NEJMc2010419 32302078 PMC7182018

[B81] Grant R. A. Morales-Nebreda L. Swaminathan S. Querrey M. Guzman E. R. Abbott D. A.(2021). Circuits Between Infected Macrophages and T Cells in SARS-CoV-2 Pneumonia. Nature 590 (7847), 635–641. 33429418 10.1038/s41586-020-03148-w PMC7987233

[B20] Guan W. J. Liang W. H. Zhao Y. Liang H. R. Chen Z. S. Li Y. M. (2020). Comorbidity and its Impact on 1590 Patients with COVID-19 in China: a Nationwide Analysis. Eur. Respir. J. 55 (5), 2000547. 10.1183/13993003.00547-2020 32217650 PMC7098485

[B21] Guan W. J. Ni Z. Y. Hu Y. Liang W. H. Ou C. Q. He J. X. (2020). Clinical Characteristics of Coronavirus Disease 2019 in China. N. Engl. J. Med. 382 (18), 1708–1720. 10.1056/NEJMoa2002032 32109013 PMC7092819

[B22] Halpin D. M. G. Criner G. J. Papi A. Singh D. Anzueto A. Martinez F. J. (2021). Global Initiative for the Diagnosis, Management, and Prevention of Chronic Obstructive Lung Disease. The 2020 GOLD Science Committee Report on COVID-19 and Chronic Obstructive Pulmonary Disease. Am. J. Respir. Crit. Care Med. 203 (1), 24–36. 10.1164/rccm.202009-3533SO 33146552 PMC7781116

[B23] Halpin D. M. G. Singh D. Hadfield R. M. (2020). Inhaled Corticosteroids and COVID-19: a Systematic Review and Clinical Perspective. Eur. Respir. J. 55 (5), 2001009. 10.1183/13993003.01009-2020 32341100 PMC7236828

[B24] Han L. Wang J. Ji X. B. Wang Z. Y. Wang Y. Zhang L. Y. (2021). Transcriptomics Analysis Identifies the Presence of Upregulated Ribosomal Housekeeping Genes in the Alveolar Macrophages of Patients with Smoking-Induced Chronic Obstructive Pulmonary Disease. Int. J. Chron. Obstruct Pulmon Dis. 16, 2653–2664. 10.2147/COPD.S313252 34588774 PMC8473850

[B25] Henrot P. Prevel R. Berger P. Dupin I. (2019). Chemokines in COPD: From Implication to Therapeutic Use. Int. J. Mol. Sci. 20 (11), 2785. 10.3390/ijms20112785 31174392 PMC6600384

[B26] Hisamoto K. Bender J. R. (2005). Vascular Cell Signaling by Membrane Estrogen Receptors. Steroids 70 (5-7), 382–387. 10.1016/j.steroids.2005.02.011 15862821

[B27] Hoffmann H. H. Sánchez-Rivera F. J. Schneider W. M. Luna J. M. Soto-Feliciano Y. M. Ashbrook A. W. (2021). Functional Interrogation of a SARS-CoV-2 Host Protein Interactome Identifies Unique and Shared Coronavirus Host Factors. Cell Host Microbe 29 (2), 267. 10.1016/j.chom.2020.12.009 33357464 PMC7833927

[B28] Horby P. Horby P. Lim W. S. Emberson J. R. Mafham M. Bell J. L. (2021). Dexamethasone in Hospitalized Patients with Covid-19. N. Engl. J. Med. 384 (8), 693–704. 10.1056/NEJMoa2021436 32678530 PMC7383595

[B29] Hou X. Zhang X. Wu X. Lu M. Wang D. Xu M. (2020). Serum Protein Profiling Reveals a Landscape of Inflammation and Immune Signaling in Early-Stage COVID-19 Infection. Mol. Cel Proteomics 19 (11), 1749–1759. 10.1074/mcp.RP120.002128 PMC7664125 32788344

[B30] Hu K. Yao L. Yan Y. Zhou L. Li J. (2021). Comprehensive Analysis of YTH Domain Family in Lung Adenocarcinoma: Expression Profile, Association with Prognostic Value, and Immune Infiltration. Dis. Markers 2021, 2789481. 10.1155/2021/2789481 34497675 PMC8420974

[B31] Hu W. P. Zeng Y. Y. Zuo Y. H. Zhang J. (2018). Identification of Novel Candidate Genes Involved in the Progression of Emphysema by Bioinformatic Methods. Int. J. Chron. Obstruct Pulmon Dis. 13, 3733–3747. 10.2147/COPD.S183100 30532529 PMC6241693

[B32] Huang J. Jiang W. Tong X. Zhang L. Zhang Y. Fan H. (2019). Identification of Gene and microRNA Changes in Response to Smoking in Human Airway Epithelium by Bioinformatics Analyses. Medicine (Baltimore) 98 (38), e17267. 10.1097/MD.0000000000017267 31568004 PMC6756728

[B33] Husebø G. R. Gabazza E. C. D'Alessandro Gabazza C. Yasuma T. Toda M. Aanerud M. (2021). Coagulation Markers as Predictors for Clinical Events in COPD. Respirology 26 (4), 342–351. 10.1111/resp.13971 33164314

[B34] Inciardi R. M. Adamo M. Lupi L. Cani D. S. Di Pasquale M. Tomasoni D. (2020). Characteristics and Outcomes of Patients Hospitalized for COVID-19 and Cardiac Disease in Northern Italy. Eur. Heart J. 41 (19), 1821–1829. 10.1093/eurheartj/ehaa388 32383763 PMC7239204

[B35] Kasahara Y. Tuder R. M. Cool C. D. Lynch D. A. Flores S. C. Voelkel N. F. (2001). Endothelial Cell Death and Decreased Expression of Vascular Endothelial Growth Factor and Vascular Endothelial Growth Factor Receptor 2 in Emphysema. Am. J. Respir. Crit. Care Med. 163 (3 Pt 1), 737–744. 10.1164/ajrccm.163.3.2002117 11254533

[B36] Kelchtermans J. Chang X. March M. E. Mentch F. Sleiman P. M. A. Hakonarson H. (2021). HIF-1α Pulmonary Phenotype Wide Association Study Unveils a Link to Inflammatory Airway Conditions. Front. Genet. 12, 756645. 10.3389/fgene.2021.756645 34621299 PMC8490729

[B37] Ko F. W. Chan K. P. Hui D. S. Goddard J. R. Shaw J. G. Reid D. W. (2016). Acute Exacerbation of COPD. Respirology 21 (7), 1152–1165. 10.1111/resp.12780 27028990 PMC7169165

[B38] Kuno T. Takahashi M. Obata R. Maeda T. (2020). Cardiovascular Comorbidities, Cardiac Injury, and Prognosis of COVID-19 in New York City. Am. Heart J. 226, 24–25. 10.1016/j.ahj.2020.05.005 32425197 PMC7227573

[B39] Lagi F. Piccica M. Graziani L. Vellere I. Botta A. Tilli M. (2020). Early Experience of an Infectious and Tropical Diseases Unit during the Coronavirus Disease (COVID-19) Pandemic, Florence, Italy, February to March 2020. Euro Surveill. 25 (17), 2000556. 10.2807/1560-7917.ES.2020.25.17.2000556 32372754 PMC7201949

[B40] Leung J. M. Niikura M. Yang C. W. T. Sin D. D. (2020). COVID-19 and COPD. Eur. Respir. J. 56 (2), 2002108. 10.1183/13993003.02108-2020 32817205 PMC7424116

[B41] Li N. Yang F. Liu D. Y. Guo J. T. Ge N. Sun S. Y. (2021). Scoparone Inhibits Pancreatic Cancer through PI3K/Akt Signaling Pathway. World J. Gastrointest. Oncol. 13 (9), 1164–1183. 10.4251/wjgo.v13.i9.1164 34616521 PMC8465440

[B42] Lian J. Jin X. Hao S. Jia H. Cai H. Zhang X. (2020). Epidemiological, Clinical, and Virological Characteristics of 465 Hospitalized Cases of Coronavirus Disease 2019 (COVID-19) from Zhejiang Province in China. Influenza Other Respir. Viruses 14 (5), 564–574. 10.1111/irv.12758 32397011 PMC7273099

[B43] Liu W. Tao Z. W. Wang L. Yuan M. L. Liu K. Zhou L. (2020). Analysis of Factors Associated with Disease Outcomes in Hospitalized Patients with 2019 Novel Coronavirus Disease. Chin. Med. J. (Engl) 133 (9), 1032–1038. 10.1097/CM9.0000000000000775 32118640 PMC7147279

[B44] Liu Y. Li S. Zhang G. Nie G. Meng Z. Mao D. (2013). Genetic Variants in IL1A and IL1B Contribute to the Susceptibility to 2009 Pandemic H1N1 Influenza A Virus. BMC Immunol. 14, 37. 10.1186/1471-2172-14-37 23927441 PMC3750637

[B45] Magro C. Mulvey J. J. Berlin D. Nuovo G. Salvatore S. Harp J. (2020). Complement Associated Microvascular Injury and Thrombosis in the Pathogenesis of Severe COVID-19 Infection: A Report of Five Cases. Transl Res. 220, 1–13. 10.1016/j.trsl.2020.04.007 32299776 PMC7158248

[B46] Mahmud S. M. H. Al-Mustanjid M. Akter F. Rahman M. S. Ahmed K. Rahman M. H. (2021). Bioinformatics and System Biology Approach to Identify the Influences of SARS-CoV-2 Infections to Idiopathic Pulmonary Fibrosis and Chronic Obstructive Pulmonary Disease Patients. Brief Bioinform 22 (5), bbab115. 10.1093/bib/bbab115 33847347 PMC8083324

[B47] Melchjorsen J. Sørensen L. N. Paludan S. R. (2003). Expression and Function of Chemokines during Viral Infections: from Molecular Mechanisms to *In Vivo* Function. J. Leukoc. Biol. 74 (3), 331–343. 10.1189/jlb.1102577 12949236 PMC7166880

[B48] Mercer P. F. Shute J. K. Bhowmik A. Donaldson G. C. Wedzicha J. A. Warner J. A. (2005). MMP-9, TIMP-1 and Inflammatory Cells in Sputum from COPD Patients during Exacerbation. Respir. Res. 6, 151. 10.1186/1465-9921-6-151 16372907 PMC1351193

[B49] Minakata Y. Nakanishi M. Hirano T. Matsunaga K. Yamagata T. Ichinose M. (2005). Microvascular Hyperpermeability in COPD Airways. Thorax 60 (10), 882. 10.1136/thx.2005.045765 PMC1747191 16055610

[B50] Mo S. Dai L. Wang Y. Song B. Yang Z. Gu W. (2021). Comprehensive Analysis of the Systemic Transcriptomic Alternations and Inflammatory Response during the Occurrence and Progress of COVID-19. Oxid Med. Cel Longev 2021, 9998697. 10.1155/2021/9998697 PMC8397550 34457122

[B51] Morrow J. D. Qiu W. Chhabra D. Rennard S. I. Belloni P. Belousov A. (2015). Identifying a Gene Expression Signature of Frequent COPD Exacerbations in Peripheral Blood Using Network Methods. BMC Med. Genomics 8, 1. 10.1186/s12920-014-0072-y 25582225 PMC4302028

[B86] Morrow J. D. Zhou X. Lao T. Jiang Z. DeMeo D. L. Cho M. H. (2017). Functional Interactors of Three Genome-Wide Association Study Genes are Differentially Expressed in Severe Chronic Obstructive Pulmonary Disease Lung Tissue. Sci. Rep. 7, 44232. 10.1038/srep44232 28287180 PMC5347019

[B87] Morrow J. D. Chase R. P. Parker M. M. Glass K. Seo M. Divo M. (2019). RNA-Sequencing Across Three Matched Tissues Reveals Shared and Tissue-Specific Gene Expression and Pathway Signatures of COPD. Respir. Res. 20 (1), 65. 10.1186/s12931-019-1032-z 30940135 PMC6446359

[B52] Obeidat M. Nie Y. Chen V. Shannon C. P. Andiappan A. K. Lee B. (2017). Network-based Analysis Reveals Novel Gene Signatures in Peripheral Blood of Patients with Chronic Obstructive Pulmonary Disease. Respir. Res. 18 (1), 72. 10.1186/s12931-017-0558-1 28438154 PMC5404332

[B85] O'Beirne S. L. Kikkers S. A. Oromendia C. Salit J. Rostmai M. R. Ballman K. V. (2020). Alveolar Macrophage Immunometabolism and Lung Function Impairment in Smoking and Chronic Obstructive Pulmonary Disease. Am. J. Respir. Crit. Care Med. 201 (6), 735–739. 10.1164/rccm.201908-1683LE 31751151 PMC7068819

[B84] Overmyer K. A. Shishkova E. Miller I. J. Balnis J. Bernstein M. N. Peters-Clarke T. M. (2021). Large-Scale Multi-omic Analysis of COVID-19 Severity. Cell Syst. 12 (1), 23–40.e7. 10.1016/j.cels.2020.10.003 33096026 PMC7543711

[B53] Palaiodimos L. Kokkinidis D. G. Li W. Karamanis D. Ognibene J. Arora S. (2020). Severe Obesity, Increasing Age and Male Sex Are Independently Associated with Worse In-Hospital Outcomes, and Higher In-Hospital Mortality, in a Cohort of Patients with COVID-19 in the Bronx, New York. Metabolism 108, 154262. 10.1016/j.metabol.2020.154262 32422233 PMC7228874

[B54] Proudfoot A. E. (2002). Chemokine Receptors: Multifaceted Therapeutic Targets. Nat. Rev. Immunol. 2 (2), 106–115. 10.1038/nri722 11910892 PMC7097668

[B88] Raman T. O'Connor T. P. Hackett N. R. Wang W. Harvey B. G. Attiyeh M. A.(2009). Quality Control in Microarray Assessment of Gene Expression in Human Airway Epithelium. BMC Genomics 10, 493. 10.1186/1471-2164-10-493 19852842 PMC2774870

[B55] Richardson S. Hirsch J. S. Narasimhan M. Crawford J. M. McGinn T. Davidson K. W. (2020). Presenting Characteristics, Comorbidities, and Outcomes Among 5700 Patients Hospitalized with COVID-19 in the New York City Area. JAMA 323 (20), 2052–2059. 10.1001/jama.2020.6775 32320003 PMC7177629

[B56] Ritchie M. E. Phipson B. Wu D. Hu Y. Law C. W. Shi W. (2015). Limma powers Differential Expression Analyses for RNA-Sequencing and Microarray Studies. Nucleic Acids Res. 43 (7), e47. 10.1093/nar/gkv007 25605792 PMC4402510

[B57] Rose C. E. Jr. Sung S. S. Fu S. M. (2003). Significant Involvement of CCL2 (MCP-1) in Inflammatory Disorders of the Lung. Microcirculation 10 (3-4), 273–288. 10.1038/sj.mn.7800193 12851645

[B58] Sabbatucci M. Covino D. A. Purificato C. Mallano A. Federico M. Lu J. (2015). Endogenous CCL2 Neutralization Restricts HIV-1 Replication in Primary Human Macrophages by Inhibiting Viral DNA Accumulation. Retrovirology 12, 4. 10.1186/s12977-014-0132-6 25608886 PMC4314729

[B59] Shaath H. Vishnubalaji R. Elkord E. Alajez N. M. (2020). Single-Cell Transcriptome Analysis Highlights a Role for Neutrophils and Inflammatory Macrophages in the Pathogenesis of Severe COVID-19. Cells 9 (11), 2374. 10.3390/cells9112374 33138195 PMC7693119

[B60] Shannon P. Markiel A. Ozier O. Baliga N. S. Wang J. T. Ramage D. (2003). Cytoscape: a Software Environment for Integrated Models of Biomolecular Interaction Networks. Genome Res. 13 (11), 2498–2504. 10.1101/gr.1239303 14597658 PMC403769

[B61] Shen W. Wang S. Wang R. Zhang Y. Tian H. Yang X. (2021). Analysis of the Polarization States of the Alveolar Macrophages in Chronic Obstructive Pulmonary Disease Samples Based on miRNA-mRNA Network Signatures. Ann. Transl Med. 9 (16), 1333. 10.21037/atm-21-3815 34532470 PMC8422127

[B82] Singh N. K. Srivastava S. Zaveri L. Bingi T. C. Mesipogu R. Kumar V. S.(2021). Host Transcriptional Response to SARS-CoV-2 Infection in COVID-19 Patients. Clin. Transl. Med. 11 (9), e534. 10.1002/ctm2.534 34586723 PMC8453261

[B62] Sun X. Gao X. Mu B. K. Wang Y. (2021). Understanding the Role of Corneal Biomechanics-Associated Genetic Variants by Bioinformatic Analyses. Int. Ophthalmol [Epub ahead of print]. 10.1007/s10792-021-02081-9 34642840

[B63] Suttorp M. M. Siegerink B. Jager K. J. Zoccali C. Dekker F. W. (2015). Graphical Presentation of Confounding in Directed Acyclic Graphs. Nephrol. Dial. Transpl. 30 (9), 1418–1423. 10.1093/ndt/gfu325 25324358

[B64] Szklarczyk D. Gable A. L. Lyon D. Junge A. Wyder S. Huerta-Cepas J. (2019). STRING V11: Protein-Protein Association Networks with Increased Coverage, Supporting Functional Discovery in Genome-wide Experimental Datasets. Nucleic Acids Res. 47 (D1), D607–D613. 10.1093/nar/gky1131 30476243 PMC6323986

[B65] Tan S. L. Ganji G. Paeper B. Proll S. Katze M. G. (2007). Systems Biology and the Host Response to Viral Infection. Nat. Biotechnol. 25 (12), 1383–1389. 10.1038/nbt1207-1383 18066032 PMC7097743

[B66] Ueland T. Holter J. C. Holten A. R. Müller K. E. Lind A. Bekken G. K. (2020). Distinct and Early Increase in Circulating MMP-9 in COVID-19 Patients with Respiratory Failure. J. Infect. 81 (3), e41–e43. 10.1016/j.jinf.2020.06.061 32603675 PMC7320854

[B67] Wei J. Alfajaro M. M. DeWeirdt P. C. Hanna R. E. Lu-Culligan W. J. Cai W. L. (2021). Genome-wide CRISPR Screens Reveal Host Factors Critical for SARS-CoV-2 Infection. Cell 184 (1), 76–e13. 10.1016/j.cell.2020.10.028 33147444 PMC7574718

[B68] Wells J. M. Gaggar A. Blalock J. E. (2015). MMP Generated Matrikines. Matrix Biol. 44-46, 122–129. 10.1016/j.matbio.2015.01.016 25636538 PMC4838901

[B69] Wilkinson T. M. Hurst J. R. Perera W. R. Wilks M. Donaldson G. C. Wedzicha J. A. (2006). Effect of Interactions between Lower Airway Bacterial and Rhinoviral Infection in Exacerbations of COPD. Chest 129 (2), 317–324. 10.1378/chest.129.2.317 16478847 PMC7094441

[B70] Xia Y. C. Radwan A. Keenan C. R. Langenbach S. Y. Li M. Radojicic D. (2017). Glucocorticoid Insensitivity in Virally Infected Airway Epithelial Cells Is Dependent on Transforming Growth Factor-β Activity. Plos Pathog. 13 (1), e1006138. 10.1371/journal.ppat.1006138 28046097 PMC5234851

[B71] Xu Z. Shi L. Wang Y. Zhang J. Huang L. Zhang C. (2020). Pathological Findings of COVID-19 Associated with Acute Respiratory Distress Syndrome. Lancet Respir. Med. 8 (4), 420–422. 10.1016/S2213-2600(20)30076-X 32085846 PMC7164771

[B72] Yan B. Freiwald T. Chauss D. Wang L. West E. Mirabelli C. (2021). SARS-CoV-2 Drives JAK1/2-dependent Local Complement Hyperactivation. Sci. Immunol. 6 (58), eabg0833. 10.1126/sciimmunol.abg0833 33827897 PMC8139422

[B73] Yan X. Li F. Wang X. Yan J. Zhu F. Tang S. (2020). Neutrophil to Lymphocyte Ratio as Prognostic and Predictive Factor in Patients with Coronavirus Disease 2019: A Retrospective Cross-Sectional Study. J. Med. Virol. 92 (11), 2573–2581. 10.1002/jmv.26061 32458459 PMC7283791

[B74] Yao Y. Wang H. Liu Z. (2020). Expression of ACE2 in Airways: Implication for COVID-19 Risk and Disease Management in Patients with Chronic Inflammatory Respiratory Diseases. Clin. Exp. Allergy 50 (12), 1313–1324. 10.1111/cea.13746 32975865 PMC7646264

[B75] Youn J. Y. Zhang Y. Wu Y. Cannesson M. Cai H. (2021). Therapeutic Application of Estrogen for COVID-19: Attenuation of SARS-CoV-2 Spike Protein and IL-6 Stimulated, ACE2-dependent NOX2 Activation, ROS Production and MCP-1 Upregulation in Endothelial Cells. Redox Biol. 46, 102099. 10.1016/j.redox.2021.102099 34509916 PMC8372492

[B76] Yu G. Wang L. G. Han Y. He Q. Y. (2012). clusterProfiler: an R Package for Comparing Biological Themes Among Gene Clusters. OMICS 16 (5), 284–287. 10.1089/omi.2011.0118 22455463 PMC3339379

[B77] Zhang W. Zhao Y. Zhang F. Wang Q. Li T. Liu Z. (2020). The Use of Anti-inflammatory Drugs in the Treatment of People with Severe Coronavirus Disease 2019 (COVID-19): The Perspectives of Clinical Immunologists from China. Clin. Immunol. 214, 108393. 10.1016/j.clim.2020.108393 32222466 PMC7102614

[B78] Zhang X. Bao W. Fei X. Zhang Y. Zhang G. Zhou X. (2018). Progesterone Attenuates Airway Remodeling and Glucocorticoid Resistance in a Murine Model of Exposing to Ozone. Mol. Immunol. 96, 69–77. 10.1016/j.molimm.2018.02.009 29501934

[B79] Zhou G. Soufan O. Ewald J. Hancock R. E. W. Basu N. Xia J. (2019). NetworkAnalyst 3.0: a Visual Analytics Platform for Comprehensive Gene Expression Profiling and Meta-Analysis. Nucleic Acids Res. 47 (W1), W234–W241. 10.1093/nar/gkz240 30931480 PMC6602507

[B80] Zhou Y. Zhou B. Pache L. Chang M. Khodabakhshi A. H. Tanaseichuk O. (2019). Metascape Provides a Biologist-Oriented Resource for the Analysis of Systems-Level Datasets. Nat. Commun. 10 (1), 1523. 10.1038/s41467-019-09234-6 30944313 PMC6447622

