# Corrigendum: Host factor interaction networks identified by integrative bioinformatics analysis reveals therapeutic implications in COPD patients with COVID-19

**DOI:** 10.3389/fphar.2023.1256022

**Published:** 2023-07-26

**Authors:** Wenjiang Zheng, Ting Wang, Peng Wu, Qian Yan, Chengxin Liu, Hui Wu, Shaofeng Zhan, Xiaohong Liu, Yong Jiang, Hongfa Zhuang

**Affiliations:** ^1^ The First Clinical Medical School, Guangzhou University of Chinese Medicine, Guangzhou, China; ^2^ The First Affiliated Hospital of Chinese Medicine, Guangzhou University of Chinese Medicine, Guangzhou, China; ^3^ Shenzhen Hospital of Integrated Traditional Chinese and Western Medicine, Shenzhen, China

**Keywords:** COPD, COVID-19, comorbidity, bioinformatics analyses, host factor interaction networks

In the published article, there was an error in the **Funding** statement. The section “the XL Famous Traditional Chinese Medicine Inheritance Studio from the Traditional Chinese Medicine Bureau of Guangdong Province (Grant No. 201805)” is incorrect. It should be “the scientific research project of Traditional Chinese Medicine Bureau of Guangdong Province (20201092).” The correct **Funding** statement appears below.

“This research was funded by grants from the “Double First-Class” And High-level University Discipline Collaborative Innovation Team Project of Guangzhou University of Chinese Medicine (Grant No.2021XK16), the Guangdong Provincial Department of Education Innovation Team Project (Grant No.2018KCXTD007), the Scientific Research Project of Guangdong Provincial Bureau of Traditional Chinese Medicine (Grant No.20212056), the Key-Area Research and Development Program of Guangdong Province (Grant No. 2020B1111100002), the National Natural Science Foundation of China (Grant Nos.81973814 and 81904132), the Natural Science Foundation of Guangdong Province (Grant No. 2017A030310129), the Natural Science Foundation of Guangdong Province (Grant No. 2020A1515010589), the scientific research project of Traditional Chinese Medicine Bureau of Guangdong Province (20201092), the Construction Project of Respiratory Department National Clinical Medical Research Center (Grant No. 2110200309), 2018 Guangzhou University of Chinese Medicine National University Student Innovation and Entrepreneurship Training Project (Grant No. 201810572038), 2020 National College Student Innovation and Entrepreneurship Training Project of Guangzhou University of Chinese Medicine (Grant No. 202010572001), the Student Learning Team Incubation Project of Innovation Academy from The First Affiliated Hospital of Guangzhou University of Chinese Medicine (Grant No. 2018XXTD003), and the Technology Research of COVID-19 Treatment and Prevention and Special Project of Traditional Chinese Medicine Application-Research on the platform construction for the prevention and treatment of viral infectious diseases with traditional Chinese medicine (Grant No. 2020KJCXKTYJ-130).”

The authors apologize for this error and state that this does not change the scientific conclusions of the article in any way. The original article has been updated.

