# Supplementary material for: Host Factor Interaction Networks Identified by Integrative Bioinformatics Analysis Reveals Therapeutic Implications in COPD Patients With COVID-19
Source: Front Pharmacol. 2021 Dec 23;12:718874. doi: 10.3389/fphar.2021.718874 (PMC8733735; doi:10.3389/fphar.2021.718874)
Supplement: Supplementary file 1 [file DataSheet1.zip › Supplementary Material/Supplementary Table S1_Gene Source.docx]

**Supplementary Table S1:** **Gene Source**

| **Source** | **Gene** |
| --- | --- |
| PubChem (COVID-19) | MPHOSPH10, FOS, CD74, HIF1A, CXCL10, TGFBR2, CAT, AIM2, TGFBR1, FCGR3A, NR1I2, CCL2, STAT1 |
| DisGeNet (COVID-19) | LEP DPP4, CD74, G6PD, LTF, HIF1A, CALCA, PPIG, LITAF, IL1RN, HP, CXCL10, SERPINE1, EGF, IL1A, FCGR3A, CCL4, CAMP, NR1I2, CCL2, STAT1, MMP9 |
| CTD (COVID-19) | MMP2, MMP7, LEP, BDNF, FOS, CD74, G6PD, CYP1A1, HIF1A, CXCL10, SERPINE1, EGF, IL1A, CAT, CXCL5, GPX2, NR1I2, CCL2, STAT1, CYP1B1, MMP9 |
| PMID:33339864(COVID-19) | PML, SELL, CCL19, TNFSF10, CD74, CXCL10, SERPINE1, CFB, AIM2, CCL4, FKBP5, GCH1, CCL2, STAT1 |
| KEGG DISEASE (COVID-19) | CXCL10, CFB, FOS, CCL2, STAT1 |
| GSE155249 (COVID-19) | MMP2, MMP7, DPP4, FKBP5, GCH1, CCL2, CD74, HIF1A, SERPINE1, CAT, CYP1B1, MMP9 |
| GSE147507 (COVID-19) | SELL, CCL19, TNFSF10, CYP1A1, PPIG, LITAF, HP, IL1A, TGFBR1, FCGR3A, CCL4, GCH1, CAMP, GPX2, CCL2, STAT1, PML, MPHOSPH10, FOS, CD74, G6PD, LTF, IL1RN, CXCL10, TGFBR2, CFB, AIM2 |
| GSE166530 (COVID-19) | LEP, BDNF, CALCA, CXCL5, NR1I2 |
| GSE157103 (COVID-19) | LEP, LTF, EGF |
| DisGeNET (COPD) | MMP2, CXCL10, SERPINE1, MMP7, TGFBR2, EGF, CAT, MPHOSPH10, AIM2, TGFBR1, DPP4, FCGR3A, FOS, TNFSF10, NR1I2, CD74, CCL2, HIF1A, CALCA, LITAF, MMP9, HP |
| CTD (COPD) | MMP2, LEP, SELL, CCL19, BDNF, FOS, TNFSF10, G6PD, CYP1A1, LTF, HIF1A, CALCA, IL1RN, CXCL10, SERPINE1, IL1A, CAT, CXCL5, CFB, TGFBR1, CCL4, FKBP5, GCH1, GPX2, CCL2, STAT1, CYP1B1, MMP9 |
| GeneCards (COPD) | CXCL10, SERPINE1, PML, IL1A, CAT, BDNF, CAMP, G6PD, CYP1A1, CCL2, PPIG, IL1RN, MMP9 |
| GSE130928(COPD) | MMP2, CXCL10, SERPINE1, MMP7, TGFBR2, IL1A, CXCL5, CFB, AIM2, TGFBR1, CCL4, GCH1, TNFSF10, CCL2, STAT1, CYP1B1, HP |
| GSE76925 (COPD) | LEP, MPHOSPH10, SELL, CCL19, DPP4, HIF1A, PPIG, CAT, FCGR3A, MMP9 |
| GSE11906(COPD) | MMP7, DPP4, BDNF, CYP1A1, CALCA, LITAF, HP, EGF, IL1A, TGFBR1, FKBP5, CAMP, GPX2, CCL2, PML, FOS, CD74, G6PD, LTF, IL1RN, NR1I2, CYP1B1 |
| GSE124180 (COPD) | CAMP |
