# Supplementary material for: Host Factor Interaction Networks Identified by Integrative Bioinformatics Analysis Reveals Therapeutic Implications in COPD Patients With COVID-19
Source: Front Pharmacol. 2021 Dec 23;12:718874. doi: 10.3389/fphar.2021.718874 (PMC8733735; doi:10.3389/fphar.2021.718874)
Supplement: Supplementary file 1 [file DataSheet1.zip › Supplementary Material/Supplementary Table S2_ TF-gene interaction.docx]

**Supplementary Table S2: TF-gene interaction**

| **Identified genes** | **Transcriptionfactors** |
| --- | --- |
| BDNF | SMC3, ZBTB17, KLF9, EHMT2, ZNF423, MAZ, EZH2, KLF8, MYNN |
| CALCA | EZH2, SUZ12, WT1, THRB, EED, CEBPA, BCL6 |
| CAT | THRB, NFE2, GATA1, DIDO1, NR2F6, ZNF197, TFDP1, RERE, DNMT1, DMAP1, MYBL2, SP1, ZNF324, IKZF1, HMG20B, CBFB, ZBTB40, KLF1, FOSL1, SOX5, GATAD2A, MBD4, ARID4B, ATF1, HNF4G} |
| CCL19 | MXD4, HMG20A |
| CCL2 | CEBPG, YY1, KLF16 |
| CCL4 | NFIC, EBF1, MTA2, ZNF366, SUPT5H |
| CD74 | PML, EED, CBFB, ATF1, MTA2, SUPT5H, RXRB, CHD7, ZNF76, MYB, TRIM22, KAT2A, TARDBP, TBX21, RFXANK, ZEB1, NFYB, POU2F2, RFX5, WHSC1, HDGF, WRNIP1, MLLT1, CEBPB |
| CFB | KLF9, THRB, NFE2, NR2F6, ZNF197, RERE, DMAP1, HMG20B, KLF1, FOSL1, GATAD2A, HMG20A, KLF16, RXRB, RFXANK, KDM5B, GATAD1, IRF1, TFAP4, KLF11, NR4A1, MXD3, ATF3, MIXL1, KDM1A, SIN3A, BCL11B, PHF8, TRIM24, MBD1, SOX13, HHEX, NR2F1, ZHX2, CUX1, FOXJ2, ZNF175, ZBTB11, MLX, TGIF2, TFE3, LEF1, GMEB2, BACH1, DRAP1, FOXK2, SAP30, KLF13, POLR2A, TEAD1, ZBTB26, RELA, HNF4A, SSRP1, ZBTB33, ZNF580, ATF4, ZNF589, GATA4 |
| CXCL10 | ZNF175 |
| CXCL5 | NFE2, ZNF584, ZNF501, ZNF24 |
| CYP1A1 | THRB, BCL6, TFDP1, ZNF76, TFAP4, SOX13, ZNF580, HBP1, ZFP2, ELF3, RAD21 |
| CYP1B1 | EZH2, TFDP1, CBFB, NFIC, KLF7, KLF4, HDAC6, PPARG, HDAC2, CTBP1, SP3 |
| DPP4 | SUZ12, NR2F6, ZNF197, RERE, HMG20B, FOSL1, SOX5, ATF1, HMG20A, KLF16, SOX13, HHEX, ZBTB26 |
| EGF | MEF2D, RFX1, GATA2 |
| FCGR3A | TFDP1, ATF1, ATF3, TRIM24, KLF13, POLR2A, RELA, MEF2D, BDP1, TAL1, ZNF558, POLR3A, SMARCE1, CREB3, BRF1, SMARCA4, SIRT6, SREBF2, DPF2, GABPA, TBP, TSHZ1, ARID1B, ZNF407, ID3, ARNT, ZNF101, PBX2 |
| FKBP5 | KLF9, MAZ, WT1, NR2F6, TFDP1, SP1, CBFB, ZBTB40, YY1, ZNF76, TBX21, HDGF, GATAD1, IRF1, KLF11, BCL11B, NR2F1, ZBTB11, TGIF2, LEF1, POLR2A, ZNF580, ZNF24, HBP1, PPARG, MEF2D, GATA2, SMARCE1, GABPA, SMAD4, HDAC1, ZNF71, ZNF382, ZNF394, RARA, RAD51, SMARCA5, ADNP, BCOR, MITF, ELF1, E2F5, TRIM28 |
| FOS | MAZ, EZH2, WT1, BCL6, GATA1, NR2F6, ZBTB40, ARID4B, ATF1, HMG20A, YY1, ZNF366, SUPT5H, MLLT1, KDM5B, MXD3, SIN3A, PHF8, CUX1, FOXJ2, ZBTB11, SAP30, TEAD1, ZNF501, ELF3, PPARG, GATA2, SREBF2, SMAD4, ZNF394, ESRRA, CHD1, STAT3, GTF2F1, BHLHE40, CCNT2, ELK1, HMGN3, CREB1, MXI1, ETS1, GATA3, NR2F2, THAP1, HCFC1, RFX3, NFIA, MBD2, MTA1, ZNF217, ZNF384, GLIS2 |
| G6PD | MAZ, TFDP1, SP1, ZNF324, IKZF1, ATF1, KLF16, ZNF76, MXD3, SIN3A, NR2F1, CUX1, TFE3, POLR2A, ELF3, HDAC6, HDAC2, ARID1B, ELF1, CHD1, CCNT2, ELK1, HMGN3, GLIS2, EGR2, ZNF143, ZNF263, KLF6, NFYC, CREM, KDM5A, IRF4, ZNF341, EGR1, FOXM1, SMAD5 |
| GCH1 | MAZ, NR2F6, CEBPG, PPARG, ZNF394, CHD1, ZNF341, SCRT2, NONO, HIC1, PRDM1 |
| GPX2 | THRB, TFDP1, MYBL2, MBD4, ARID4B, ATF1, HNF4G, CEBPG, KLF16, RFXANK, KLF11, MXD3, SOX13, NR2F1, TGIF2, SSRP1, ZNF580, ETV4, RCOR2 |
| HIF1A | EHMT2, MAZ, ATF1, ZNF76, MLLT1, FOXJ2, GABPA, CHD1, ZNF644, ZBTB7A, USF2, RBBP5 |
| HP | KLF9, THRB, ZNF197, RERE, HMG20B, FOSL1, GATAD2A, ARID4B, MXD4, KLF11, ATF3, MBD1, SOX13, NR2F1, MLX, TFE3, SSRP1, GATA4, ELF3, ZNF7 |
| IL1A | TFDP1, MYBL2, MBD4, HNF4G, KLF16, IRF1, FOXJ2, ID3, ADNP, ELF1, KLF6, TCF7 |
| IL1RN | ARID4B, ATF1, MXD4, KLF16, RXRB, GATAD1, TFAP4, ATF3, DRAP1, ZBTB26, SSRP1, ZNF580, ZNF589, HBP1, RAD21, HDAC6, HDAC2, GABPA, PBX2, SMAD4, RARA, ETV4, FOSL2 |
| LEP | ATF1, GATA2, RAD51, GTF2E2 |
| LITAF | KLF9, MAZ, EED, NR2F6, ZNF197, TFDP1, RERE, DMAP1, HMG20B, FOSL1, SOX5, HMG20A, CEBPG, RXRB, RFXANK, WRNIP1, MLLT1, KDM5B, KLF11, MXD3, KDM1A, PHF8, SOX13, HHEX, TGIF2, DRAP1, SAP30, ZBTB26, ZBTB33, ZNF580, ATF4, GATA4, RAD21, SMAD4, RARA, SMARCA5, ADNP, E2F5, RFX3, NFIA, ETV4, ZNF644, RNF2, FOXA3, TEAD3, L3MBTL2, PRDM10, NFIL3, ZFP64 |
| MMP2 | EZH2, ETS1, NR2F2, THAP1 |
| MMP7 | NFIC, EBF1，SUPT5H |
| MMP9 | MAZ, EZH2, MXD4, KLF16, RFXANK, GATAD1, TFAP4, MXD3, KDM1A, HHEX, CUX1, DRAP1, ZBTB26, SSRP1, ID3, MTA1, KDM5A, ZNF341, ZNF7, CTCF, CTBP2, ZKSCAN1 |
| MPHOSPH10 | KLF9, ARID4B, HMG20A, CEBPG, KLF11, SIN3A, SOX13, ZHX2, TFE3, SSRP1, ZNF501, RAD21, PPARG, ARID1B, SMAD4, ADNP, CHD1, GTF2F1, CREB1, NFIA, ZNF217, KDM5A, SMAD5, ZNF644, GTF2E2, ZFP64, CREB3L1, MYC, TAF7, TCF7L2, ZFP37, NRF1, NFRKB |
| NR1I2 | DMAP1, ELF3, FOXA3 |
| PML | MAZ, KLF8, SP1, CBFB, KLF1, RXRB, ZNF76, NR4A1, BCL11B, POLR2A, GATA2, SMAD4, ZNF382, ELK1, ETS1, IRF4, EGR1, FOXM1, ZFP37, IRF2, RUNX3, PRDM2, ZFX, SPI1, ZNF610, GFI1B |
| PPIG | THRB, SP1, HMG20B, ATF1, TARDBP, RFXANK, ZEB1, KDM5B, IRF1, KLF11, PHF8, NR2F1, ZHX2, GMEB2, SAP30, KLF13, ZNF24, PPARG, ARID1B, ARNT, ZNF394, SMARCA5, BCOR, ELF1, E2F5, ETS1, HCFC1, IRF4, EGR1, FOXM1, SMAD5, ZNF644, GTF2E2, CTCF, CREB3L1, MYC, IRF2, NCOA1, POLR2H, ZNF264, JUND, NFE2L1, ZNF512, THRAP3, HDAC6, HDAC2 |
| SELL | HDAC6, HDAC2 |
| SERPINE1 | SMC3, EED, BCL6, ZNF197, RERE, DMAP1, HMG20B, FOSL1, ARID4B, MXD4, HMG20A, KLF16, ZEB1, GATAD1, IRF1, KDM1A, SIN3A, SOX13, NR2F1, ZNF175, MLX, TGIF2, TFE3, SSRP1, ZBTB33, ZNF580, ZNF589, GATA4, ZNF584, HDAC6, PPARG, HDAC2, MEF2D, DPF2, ZNF394, SMARCA5, ADNP, BCOR, E2F5, MXI1, ZNF384, IRF4, EGR1, FOXM1, SMAD5, HIC1, RCOR2, ZBTB7A, ZNF7, FOSL2, ZFP37, NRF1, NFRKB, TBX3, SP7, HLF, ZNF639 |
| TGFBR1 | SIN3A, RELA, ZNF24, CREB3, GABPA, ARID1B, CREM, IRF4, EGR1, FOXM1, ZBTB7A, ZFX |
| TGFBR2 | ZNF197, RERE, FOSL1, SOX5, GATAD2A, ARID4B, CEBPG, SUPT5H, RFXANK, HHEX, TGIF2, TFE3, ZBTB26, SSRP1, HBP1, ZNF382, ZNF644, FOSL2, L3MBTL2, JUND |
| TNFSF10 | TFDP1, CEBPB, SOX13, CREB1, HMBOX1 |
