# Supplementary material for: Host Factor Interaction Networks Identified by Integrative Bioinformatics Analysis Reveals Therapeutic Implications in COPD Patients With COVID-19
Source: Front Pharmacol. 2021 Dec 23;12:718874. doi: 10.3389/fphar.2021.718874 (PMC8733735; doi:10.3389/fphar.2021.718874)
Supplement: Supplementary file 1 [file DataSheet1.zip › Supplementary Material/Supplementary Table S3_ Gene-miRNA interaction.docx]

| **Supplementary Table S3: Gene-miRNA interaction** | |
| --- | --- |
| **Identified genes** | **miRNA** |
| NR1I2 | hsa-let-7a-5p, hsa-mir-16-5p, hsa-mir-18a-5p, hsa-mir-148a-3p, hsa-mir-615-3p, hsa-mir-3c-1-3p |
| HIF1A | hsa-let-7b-5p, hsa-mir-17-5p, hsa-mir-18a-5p, hsa-mir-2a-5p, hsa-mir-21-5p, hsa-mir-22-3p, hsa-mir-27a-3p, hsa-mir-33a-5p, hsa-mir-93-5p, hsa-mir-16a-5p, hsa-mir-17, hsa-mir-199a-5p, hsa-mir-199b-5p, hsa-mir-21-3p, hsa-mir-217, hsa-mir-138-5p, hsa-mir-142-5p, hsa-mir-145-5p, hsa-mir-186-5p, hsa-mir-155-5p, hsa-mir-16b-5p, hsa-mir-34b-5p, hsa-mir-151a-3p, hsa-mir-338-3p, hsa-mir-424-5p, hsa-mir-18b-5p, hsa-mir-2b-5p, hsa-mir-429, hsa-mir-433-3p, hsa-mir-494-3p, hsa-mir-519c-3p, hsa-mir-519d-3p, hsa-mir-299-5p, hsa-mir-558, hsa-mir-589-3p, hsa-mir-16a-3p, hsa-mir-138-1-3p, hsa-mir-2c-5p, hsa-mir-16b-3p, hsa-mir-497-3p, hsa-mir-59-5p, hsa-mir-576-3p, hsa-mir-625-3p, hsa-mir-889-3p, hsa-mir-59-3-5p, hsa-mir-935, hsa-mir-94, hsa-mir-1322, hsa-mir-449c-5p, hsa-mir-2682-5p, hsa-mir-3121-3p, hsa-mir-3128, hsa-mir-374-3p, hsa-mir-316-3p, hsa-mir-4282, hsa-mir-369, hsa-mir-3662, hsa-mir-3668, hsa-mir-4418, hsa-mir-548ah-5p, hsa-mir-4463, hsa-mir-4464, hsa-mir-447, hsa-mir-4487, hsa-mir-4499, hsa-mir-4662a-5p, hsa-mir-4735-3p, hsa-mir-4748, hsa-mir-4799-5p, hsa-mir-594, hsa-mir-5681b, hsa-mir-5692a, hsa-mir-589-3p, hsa-mir-6511a-5p, hsa-mir-153-5p, hsa-mir-329-5p, hsa-mir-191-3p, hsa-mir-6755-5p, hsa-mir-686-5p, hsa-mir-687-3p, hsa-mir-688-5p, hsa-mir-6815-3p, hsa-mir-6893-5p, hsa-mir-855 |
| TGFBR1 | hsa-let-7b-5p, hsa-let-7c-5p, hsa-let-7d-5p, hsa-let-7f-5p, hsa-mir-2a-5p, hsa-mir-27a-3p, hsa-mir-98-5p, hsa-mir-11-3p, hsa-mir-199a-5p, hsa-mir-181a-5p, hsa-mir-181c-5p, hsa-mir-24-5p, hsa-let-7g-5p, hsa-mir-27b-3p, hsa-mir-128-3p, hsa-mir-13a-3p, hsa-mir-14-5p, hsa-mir-142-3p, hsa-mir-376c-3p, hsa-mir-379-5p, hsa-mir-135b-5p, hsa-mir-49-3p, hsa-mir-22-5p, hsa-mir-1283, hsa-mir-466, hsa-mir-3153, hsa-mir-3156-5p, hsa-mir-3681-3p, hsa-mir-3927-3p, hsa-mir-448, hsa-mir-4643, hsa-mir-4646-5p, hsa-mir-4668-5p, hsa-mir-1343-3p, hsa-mir-3529-5p, hsa-mir-4789-3p, hsa-mir-24-3p, hsa-mir-216a-3p, hsa-mir-939-3p, hsa-mir-6733-5p, hsa-mir-6739-5p, hsa-mir-6742-3p, hsa-mir-6783-3p, hsa-mir-6795-3p, hsa-mir-6826-3p, hsa-mir-6831-5p, hsa-mir-6852-5p, hsa-mir-6887-3p, hsa-mir-6888-3p, hsa-mir-863, hsa-mir-883 |
| MMP9 | hsa-let-7e-5p, hsa-mir-21-5p, hsa-mir-29b-3p, hsa-mir-24-5p, hsa-mir-211-5p, hsa-mir-15b-5p, hsa-mir-132-3p, hsa-mir-143-3p, hsa-mir-9-5p, hsa-mir-9-3p, hsa-mir-32a, hsa-mir-32a-5p, hsa-mir-338-3p, hsa-mir-133b, hsa-mir-451a, hsa-mir-491-5p, hsa-mir-524-5p, hsa-mir-892b, hsa-mir-133a-5p, hsa-mir-942-3p, hsa-mir-23a-5p |
| PPIG | hsa-let-7e-5p, hsa-mir-15a-5p, hsa-mir-16-5p, hsa-mir-13a-3p, hsa-mir-17, hsa-mir-15b-5p, hsa-mir-13a-3p, hsa-mir-142-5p, hsa-mir-186-5p, hsa-mir-195-5p, hsa-mir-31a-3p, hsa-mir-13b-3p, hsa-mir-424-5p, hsa-mir-497-5p, hsa-mir-519c-3p, hsa-mir-519b-3p, hsa-mir-525-3p, hsa-mir-519a-3p, hsa-mir-573, hsa-mir-454-3p, hsa-mir-34-5p, hsa-mir-31b-3p, hsa-mir-3133, hsa-mir-3161, hsa-mir-4295, hsa-mir-431, hsa-mir-3616-5p, hsa-mir-3666, hsa-mir-3129-3p, hsa-mir-4738-3p, hsa-mir-4795-5p, hsa-mir-5583-5p, hsa-mir-559-3p, hsa-mir-657-5p, hsa-mir-51-3p, hsa-mir-58-5p, hsa-mir-6838-5p, hsa-mir-7157-5p |
| BDNF | hsa-mir-15a-5p, hsa-mir-16-5p, hsa-mir-22-3p, hsa-mir-3a-5p, hsa-mir-96-5p, hsa-mir-1a-5p, hsa-mir-182-5p, hsa-mir-24-5p, hsa-mir-21-3p, hsa-mir-1-3p, hsa-mir-124-3p, hsa-mir-132-3p, hsa-mir-26, hsa-mir-613, hsa-mir-1-5p |
| CXCL10 | hsa-mir-15a-5p, hsa-mir-21-5p, hsa-mir-32a-5p, hsa-mir-52g-3p, hsa-mir-52h, hsa-mir-55a-3p, hsa-mir-612, hsa-mir-632, hsa-mir-661, hsa-mir-767-5p, hsa-mir-196a-3p, hsa-mir-2c-5p, hsa-mir-1226-3p, hsa-mir-1231, hsa-mir-1285-3p, hsa-mir-365-5p, hsa-mir-4288, hsa-mir-369, hsa-mir-3664-5p, hsa-mir-3672, hsa-mir-548ah-5p, hsa-mir-4522, hsa-mir-312-5p, hsa-mir-3187-5p, hsa-mir-5189-5p, hsa-mir-873-3p, hsa-mir-939-3p, hsa-mir-6849-3p, hsa-mir-686, hsa-mir-6864-3p, hsa-mir-6888-3p |
| LITAF | hsa-mir-15a-5p, hsa-mir-16-5p, hsa-mir-27a-3p, hsa-mir-7-5p, hsa-mir-15b-5p, hsa-mir-124-3p, hsa-mir-128-3p, hsa-mir-195-5p, hsa-mir-32a, hsa-mir-34b-5p, hsa-mir-375, hsa-mir-617, hsa-mir-127-5p, hsa-mir-936, hsa-mir-1287-5p, hsa-mir-1255a, hsa-mir-1255b-5p, hsa-mir-449c-5p, hsa-mir-2682-5p, hsa-mir-32e, hsa-mir-3714, hsa-mir-4484, hsa-mir-548an, hsa-mir-3978, hsa-mir-4712-3p, hsa-mir-6134, hsa-mir-3928-5p, hsa-mir-6744-5p, hsa-mir-6768-3p, hsa-mir-686-3p, hsa-mir-7151-5p, hsa-mir-883 |
| MMP2 | hsa-mir-17-5p, hsa-mir-21-5p, hsa-mir-29a-3p, hsa-mir-29b-3p, hsa-mir-218-5p, hsa-mir-221-3p, hsa-mir-125b-5p, hsa-mir-143-3p, hsa-mir-9-5p, hsa-mir-16b-5p, hsa-mir-29c-3p, hsa-mir-32a-5p, hsa-mir-13b-3p, hsa-mir-338-3p, hsa-mir-335-5p, hsa-mir-451a, hsa-mir-452-5p, hsa-mir-491-5p, hsa-mir-524-5p, hsa-mir-519d-3p, hsa-mir-52g-3p, hsa-mir-544a, hsa-mir-767-5p, hsa-mir-29b-2-5p, hsa-mir-78-5p, hsa-mir-1238-3p, hsa-mir-4691-5p, hsa-mir-67-3p, hsa-mir-6749-3p, hsa-mir-6792-3p, hsa-mir-23a-5p |
| TGFBR2 | hsa-mir-17-5p, hsa-mir-18a-5p, hsa-mir-19a-3p, hsa-mir-19b-3p, hsa-mir-2a-5p, hsa-mir-21-5p, hsa-mir-92a-3p, hsa-mir-93-5p, hsa-mir-11-3p, hsa-mir-16a-5p, hsa-mir-196a-5p, hsa-mir-147a, hsa-mir-34a-5p, hsa-mir-181c-5p, hsa-mir-24-5p, hsa-mir-211-5p, hsa-mir-216a-5p, hsa-mir-13a-3p, hsa-mir-142-5p, hsa-mir-145-5p, hsa-mir-9-5p, hsa-mir-16b-5p, hsa-mir-34b-5p, hsa-mir-34c-5p, hsa-mir-31a-3p, hsa-mir-13b-3p, hsa-mir-32b-3p, hsa-mir-32d-3p, hsa-mir-37-3p, hsa-mir-372-3p, hsa-mir-373-3p, hsa-mir-335-5p, hsa-mir-196b-5p, hsa-mir-2b-5p, hsa-mir-449a, hsa-mir-49-3p, hsa-mir-22-5p, hsa-mir-495-3p, hsa-mir-515-5p, hsa-mir-519e-5p, hsa-mir-519c-3p, hsa-mir-52a-3p, hsa-mir-526b-3p, hsa-mir-519b-3p, hsa-mir-519d-3p, hsa-mir-519a-3p, hsa-mir-55-3p, hsa-mir-57-3p, hsa-mir-582-5p, hsa-mir-59-5p, hsa-mir-595, hsa-mir-66, hsa-mir-61, hsa-mir-548c-3p, hsa-mir-63, hsa-mir-449b-5p, hsa-mir-655-3p, hsa-mir-454-3p, hsa-let-7f-2-3p, hsa-mir-33a-3p, hsa-mir-23b-5p, hsa-mir-488-3p, hsa-mir-551b-5p, hsa-mir-574-5p, hsa-mir-31b-3p, hsa-mir-94, hsa-mir-12, hsa-mir-1253, hsa-mir-1276, hsa-mir-1827, hsa-mir-449c-5p, hsa-mir-2682-5p, hsa-mir-544b, hsa-mir-548u, hsa-mir-365-5p, hsa-mir-4295, hsa-mir-4316, hsa-mir-4324, hsa-mir-32-5p, hsa-mir-369, hsa-mir-3666, hsa-mir-4438, hsa-mir-548ah-5p, hsa-mir-3978, hsa-mir-4649-3p, hsa-mir-4732-5p, hsa-mir-4796-3p, hsa-mir-595, hsa-mir-559-3p, hsa-mir-5688, hsa-mir-1185-2-3p, hsa-mir-1185-1-3p, hsa-mir-6511a-5p, hsa-mir-191-3p, hsa-mir-6758-5p, hsa-mir-6758-3p, hsa-mir-677-5p, hsa-mir-6777-3p, hsa-mir-686-5p, hsa-mir-688-5p, hsa-mir-6818-5p, hsa-mir-6832-5p, hsa-mir-6856-5p, hsa-mir-6867-5p, hsa-mir-6893-5p, hsa-mir-7151-3p, hsa-mir-7159-5p, hsa-mir-7161-5p, hsa-mir-7162-3p, hsa-mir-7849-3p |
| GPX2 | hsa-mir-17-3p, hsa-mir-335-5p |
| GCH1 | hsa-mir-18a-5p, hsa-mir-26b-5p, hsa-mir-192-5p, hsa-mir-215-5p, hsa-mir-1-3p, hsa-mir-124-3p, hsa-mir-335-5p, hsa-mir-494-3p, hsa-mir-49-5p, hsa-mir-134-5p, hsa-mir-1827, hsa-mir-482-3p, hsa-mir-942-3p, hsa-mir-6765-5p, hsa-mir-855 |
| FKBP5 | hsa-mir-21-5p, hsa-mir-22-3p, hsa-mir-28-5p, hsa-mir-99a-5p, hsa-mir-1-5p, hsa-mir-2b-3p, hsa-mir-2c-3p, hsa-mir-32c-5p, hsa-mir-328-3p, hsa-mir-429, hsa-mir-511-5p, hsa-mir-512-5p, hsa-mir-51-5p, hsa-mir-539-5p, hsa-mir-548c-3p, hsa-mir-65, hsa-mir-765, hsa-mir-23a-5p, hsa-mir-93-3p, hsa-mir-23b-5p, hsa-mir-377-5p, hsa-mir-455-3p, hsa-mir-78-5p, hsa-mir-1243, hsa-mir-1281, hsa-mir-1321, hsa-mir-1827, hsa-mir-548s, hsa-mir-3139, hsa-mir-3166, hsa-mir-3169, hsa-mir-319-5p, hsa-mir-4257, hsa-mir-427, hsa-mir-4272, hsa-mir-3612, hsa-mir-3613-3p, hsa-mir-3654, hsa-mir-3675-3p, hsa-mir-3926, hsa-mir-4419a, hsa-mir-4441, hsa-mir-4443, hsa-mir-451, hsa-mir-4537, hsa-mir-3664-3p, hsa-mir-4666a-5p, hsa-mir-4684-5p, hsa-mir-1343-3p, hsa-mir-4739, hsa-mir-474-5p, hsa-mir-4756-5p, hsa-mir-4779, hsa-mir-2467-3p, hsa-mir-4786-5p, hsa-mir-5186, hsa-mir-5589-5p, hsa-mir-569, hsa-mir-66-3p, hsa-mir-55b-2-5p, hsa-mir-939-3p, hsa-mir-686, hsa-mir-6127, hsa-mir-6129, hsa-mir-613, hsa-mir-6133, hsa-mir-6499-3p, hsa-mir-6511a-5p, hsa-mir-191-3p, hsa-mir-6742-3p, hsa-mir-6754-5p, hsa-mir-676-5p, hsa-mir-6771-3p, hsa-mir-6776-5p, hsa-mir-6783-3p, hsa-mir-6791-3p, hsa-mir-6797-5p, hsa-mir-6816-3p, hsa-mir-6829-3p, hsa-mir-6849-3p, hsa-mir-6852-5p, hsa-mir-6878-5p, hsa-mir-716-5p, hsa-mir-6516-5p, hsa-mir-88, hsa-mir-7977, hsa-mir-1249-5p, hsa-mir-4485-5p |
| CCL2 | hsa-mir-24-3p, hsa-mir-26b-5p, hsa-mir-98-5p, hsa-mir-1-3p, hsa-mir-124-3p, hsa-mir-26, hsa-mir-155-5p, hsa-mir-495-3p, hsa-let-7g-3p, hsa-mir-518a-5p, hsa-mir-24-3p, hsa-mir-195-5p |
| TNFSF10 | hsa-mir-25-3p, hsa-mir-26a-5p, hsa-mir-26b-5p, hsa-mir-98-5p, hsa-mir-221-3p, hsa-mir-222-3p, hsa-mir-562, hsa-mir-513c-5p, hsa-mir-1297, hsa-mir-365a-5p, hsa-mir-3199, hsa-mir-514b-5p, hsa-mir-4465, hsa-mir-3152-5p, hsa-mir-5697, hsa-mir-365b-5p, hsa-mir-6819-3p, hsa-mir-6836-3p, hsa-mir-6877-3p, hsa-mir-852 |
| G6PD | hsa-mir-26b-5p, hsa-mir-1-3p, hsa-mir-26, hsa-mir-335-5p, hsa-mir-92b-3p, hsa-mir-571, hsa-mir-654-5p, hsa-mir-92a-2-5p, hsa-mir-541-3p, hsa-mir-132, hsa-mir-4298, hsa-mir-4283, hsa-mir-3678-3p, hsa-mir-4648, hsa-mir-4685-5p, hsa-mir-2467-3p, hsa-mir-54-3p, hsa-mir-6769a-5p, hsa-mir-6798-5p, hsa-mir-685-5p, hsa-mir-6837-5p, hsa-mir-6846-5p, hsa-mir-6848-5p, hsa-mir-6769b-5p, hsa-mir-7113-5p, hsa-mir-889 |
| CYP1B1 | hsa-mir-27a-3p, hsa-mir-221-3p, hsa-mir-27b-3p, hsa-mir-124-3p, hsa-mir-141-3p, hsa-mir-2a-3p, hsa-mir-511-5p, hsa-mir-513a-5p, hsa-mir-148a-5p, hsa-mir-181a-2-3p, hsa-mir-187-5p, hsa-mir-2c-5p, hsa-mir-34-5p, hsa-mir-148b-5p, hsa-mir-132, hsa-mir-3148, hsa-mir-3192-5p, hsa-mir-4298, hsa-mir-4314, hsa-mir-4484, hsa-mir-548an, hsa-mir-3976, hsa-mir-4646-5p, hsa-mir-57-5p, hsa-mir-24-3p, hsa-mir-1273g-3p, hsa-mir-656-5p, hsa-mir-659-3p, hsa-mir-28a-5p, hsa-mir-619-5p, hsa-mir-627-3p, hsa-mir-28b-5p, hsa-mir-548e-5p, hsa-mir-676-3p, hsa-mir-6768-3p, hsa-mir-683-3p, hsa-mir-6874-3p, hsa-mir-6885-3p |
| CXCL5 | hsa-mir-28-5p, , hsa-mir-7-5p, , hsa-mir-186-5p, , hsa-mir-369-3p, , hsa-mir-374a-5p, , hsa-mir-148b-3p, , hsa-mir-384, , hsa-mir-41-3p, , hsa-mir-511-5p, , hsa-mir-495-3p, , hsa-mir-578, , hsa-mir-656-3p, , hsa-mir-769-5p, , hsa-mir-765, , hsa-mir-93-3p, , hsa-mir-155-3p, , hsa-mir-616-3p, , hsa-mir-78-5p, , hsa-mir-374b-5p, , hsa-mir-942-5p, , hsa-mir-1237-3p, , hsa-mir-1248, , hsa-mir-548m, , hsa-mir-1321, , hsa-mir-3133, , hsa-mir-3139, , hsa-mir-365-5p, , hsa-mir-4284, , hsa-mir-3655, , hsa-mir-3685, , hsa-mir-3692-5p, , hsa-mir-3919, , hsa-mir-4464, , hsa-mir-3124-3p, , hsa-mir-4729, , hsa-mir-4739, , hsa-mir-4748, , hsa-mir-4756-5p, , hsa-mir-4756-3p, , hsa-mir-511-5p, , hsa-mir-587, , hsa-mir-5582-3p, , hsa-mir-5692c, , hsa-mir-5688, , hsa-mir-5696, , hsa-mir-5692b, , hsa-mir-1277-5p, , hsa-mir-3191-5p, , hsa-mir-3529-3p, , hsa-mir-671, , hsa-mir-19a-3p, , hsa-mir-6728-3p, , hsa-mir-674-3p, , hsa-mir-6868-3p, , hsa-mir-716-5p, , hsa-mir-863 |
| FOS | hsa-mir-29a-3p, hsa-mir-11-3p, hsa-mir-29b-3p, hsa-mir-192-5p, hsa-mir-139-5p, hsa-mir-7-5p, hsa-mir-34a-5p, hsa-mir-181a-5p, hsa-mir-181b-5p, hsa-mir-215-5p, hsa-mir-221-3p, hsa-mir-222-3p, hsa-mir-146a-5p, hsa-mir-155-5p, hsa-mir-29c-3p, hsa-mir-338-3p, hsa-mir-335-5p, hsa-mir-196b-5p, hsa-mir-449a, hsa-mir-493-5p, hsa-mir-627-5p, hsa-mir-77-5p, hsa-mir-19a-5p, hsa-mir-19b-1-5p, hsa-mir-19b-2-5p, hsa-mir-11-5p, hsa-mir-187-5p, hsa-mir-49-5p, hsa-mir-543, hsa-mir-1234-3p, hsa-mir-548v, hsa-mir-323b-3p, hsa-mir-365-3p, hsa-mir-3622b-5p, hsa-mir-4438, hsa-mir-453, hsa-mir-464-5p, hsa-mir-4726-5p, hsa-mir-4733-3p, hsa-mir-595, hsa-mir-5581-3p, hsa-mir-5586-5p, hsa-mir-937-5p, hsa-mir-1292-3p, hsa-mir-589-3p, hsa-mir-677, hsa-mir-654-3p, hsa-mir-658-3p, hsa-mir-619-5p, hsa-mir-6816-3p, hsa-mir-6854-3p, hsa-mir-6872-3p, hsa-mir-6885-3p, hsa-mir-717-5p, hsa-mir-7151-3p, hsa-mir-88, hsa-mir-881, hsa-mir-883 |
| CAT | hsa-mir-3a-5p, hsa-mir-181b-5p, hsa-mir-3b-5p, hsa-mir-155-5p |
| IL1A | hsa-mir-3a-5p, hsa-mir-3c-5p, hsa-mir-3d-5p, hsa-mir-181a-5p, hsa-mir-181b-5p, hsa-mir-181c-5p, hsa-mir-3b-5p, hsa-mir-122-5p, hsa-mir-142-3p, hsa-mir-191-5p, hsa-mir-3e-5p, hsa-mir-335-5p, hsa-mir-181d-5p, hsa-mir-92a-1-5p |
| SERPINE1 | hsa-mir-3a-5p, hsa-mir-99a-5p, hsa-mir-192-5p, hsa-mir-148a-3p, hsa-mir-3c-5p, hsa-mir-3d-5p, hsa-mir-1a-5p, hsa-mir-24-5p, hsa-mir-224-5p, hsa-mir-3b-5p, hsa-mir-124-3p, hsa-mir-138-5p, hsa-mir-143-3p, hsa-mir-145-5p, hsa-mir-31a-3p, hsa-mir-3e-5p, hsa-mir-486-5p, hsa-mir-1273d, hsa-mir-3145-5p, hsa-mir-3977, hsa-mir-519d-5p, hsa-mir-6882-5p |
| CFB | hsa-mir-98-5p, hsa-mir-335-5p, hsa-mir-21-5p |
| MMP7 | hsa-mir-148a-3p, hsa-mir-34a-5p, hsa-mir-126-5p, hsa-mir-126-3p, hsa-mir-489-3p, hsa-mir-543, hsa-mir-23a-5p |
| STAT1 | hsa-mir-34a-5p, hsa-mir-23a-3p, hsa-mir-223-3p, hsa-mir-14-5p, hsa-mir-145-5p, hsa-mir-146a-5p, hsa-mir-15-5p, hsa-mir-155-5p, hsa-mir-45a-5p, hsa-mir-51-5p, hsa-mir-65-5p, hsa-mir-615-3p, hsa-mir-653-5p, hsa-mir-5a-5p, hsa-mir-1183, hsa-mir-4693-5p, hsa-mir-23b-3p, hsa-mir-59-3p, hsa-mir-7158-3p |
| LTF | hsa-mir-214-3p, hsa-mir-412-3p, hsa-mir-942-5p, hsa-mir-3664-5p, hsa-mir-4768-5p, hsa-mir-4778-5p, hsa-mir-3191-5p, hsa-mir-4743-3p, hsa-mir-6728-3p, hsa-mir-674-3p, hsa-mir-6754-3p, hsa-mir-689-3p, hsa-mir-6817-3p, hsa-mir-6833-3p, hsa-mir-6837-3p, hsa-mir-6845-3p, hsa-mir-6873-3p, hsa-mir-711-3p |
| CYP1A1 | hsa-mir-2b-3p, hsa-mir-2c-3p, hsa-mir-155-5p, hsa-mir-429, hsa-mir-452-5p, hsa-mir-632, hsa-mir-655-3p, hsa-mir-296-3p, hsa-mir-1231, hsa-mir-4294, hsa-mir-4288, hsa-mir-3664-5p, hsa-mir-374c-5p, hsa-mir-312-5p, hsa-mir-4676-3p, hsa-mir-4677-3p, hsa-mir-4679, hsa-mir-468-5p, hsa-mir-4768-5p, hsa-mir-3191-5p, hsa-mir-892c-3p, hsa-mir-689-3p, hsa-mir-6833-3p, hsa-mir-6888-3p, hsa-mir-884 |
| AIM2 | hsa-mir-1-3p |
| CAMP | hsa-mir-124-3p |
| MPHOSPH10 | hsa-mir-124-3p, hsa-mir-92b-3p |
| CCL19 | hsa-mir-9-5p, hsa-mir-148b-3p, hsa-mir-335-5p |
| PML | hsa-mir-378a-3p, hsa-mir-335-5p, hsa-mir-423-3p |
| EGF | hsa-mir-485-5p, hsa-mir-223-5p, hsa-mir-665, hsa-mir-94, hsa-mir-1225-3p, hsa-mir-1233-3p, hsa-mir-134-5p, hsa-mir-1827, hsa-mir-3188, hsa-mir-1273e, hsa-mir-4433a-3p, hsa-mir-4459, hsa-mir-3975, hsa-mir-4649-3p, hsa-mir-4722-5p, hsa-mir-2467-5p, hsa-mir-6765-5p, hsa-mir-687-5p, hsa-mir-688-5p, hsa-mir-684-3p, hsa-mir-6884-5p, hsa-mir-6893-5p, hsa-mir-6894-5p |
| LEP | hsa-mir-208a-3p, hsa-mir-499a-5p, hsa-mir-208b-3p, hsa-mir-3658, hsa-mir-7856-5p |
